# Evaluation of the histopathological extent of neoplastic infiltration in intestinal tumours in cats

**DOI:** 10.1002/vms3.166

**Published:** 2019-03-11

**Authors:** Michael Morrice, Gerry Polton, Sam Beck

**Affiliations:** ^1^ Maven Veterinary Care London UK; ^2^ North Downs Specialist Referrals Bletchingly UK; ^3^ Bridge Pathology Ltd. Bristol United Kingdom

**Keywords:** feline, intestine, margin, surgery, tumour, lymphoma, carcinoma

## Abstract

With the exception of intestinal lymphoma, surgery is the most commonly recommended treatment for solitary feline intestinal tumours. However, there is a lack of evidence to substantiate resection margin recommendations for these tumours. The aim of this study was to add knowledge concerning resection margins for discrete intestinal masses in cats. Thirty confirmed feline intestinal tumours removed at veterinary centres across the UK from March 2017 to March 2018 underwent histological assessment at the palpable edge of the intestinal tumour and then at every 1 cm increment to the surgeon‐cut tissue border in oral, aboral and mesenteric directions. Histological margin recommendations were developed for carcinoma and lymphoma tumour types and non‐lymphoma intestinal tumours collectively. Seventeen intestinal lymphomas, nine carcinomas, two sarcomas and two mast cell tumours were evaluated in this study. Seven of the nine intestinal carcinomas would have been completely removed with histological margins of 4 cm in oral and aboral directions. Both sarcomas and one mast cell tumour would have been removed in their entirety with 4 cm histological margins in oral and aboral directions. There was extensive and varied microscopic invasion of intestinal tissue away from discrete intestinal lymphomas in the majority of the cases in this study. There is increasing evidence in veterinary as well as human literature supporting the role of surgical resection in the treatment of discrete intestinal lymphoma. If surgery is to be considered this study supports the removal of the gross tumour only. A histological margin of 4 cm should be considered, where possible, for intestinal masses other than lymphomas.

## Introduction

Neoplasia of the gastrointestinal tract in cats is uncommon, accounting for approximately 2% to 13.5% of all feline neoplasms (Bastianello 1983; Marks 1996)_._ Lymphoma, carcinoma and mast cell tumours are the most common feline intestinal tumours (Barrs & Beatty 2012; Thamm 2015; Suwa & Shimoda 2017) with lymphoma the most common (55%) followed by carcinoma (32%) then mast cell tumours (4%) (Rissetto *et al*. 2011).

The therapy of choice for malignant intestinal non‐lymphoma tumours is excision (Marconato & Bettini 2013). It is well established that clean margins after resection of an intestinal mass result in better outcome (Bakaeen *et al*. 2000). To obtain complete excision of intestinal tumours in cats, surgical margins from as narrow as 2 cm to as wide as 8 cm have been recommended (White 2003; North & Banks 2009; Bray 2011; Marconato & Bettini 2013). However, there is a lack of clinical data and research to substantiate these recommendations (Shales 2015).

It is widely accepted that chemotherapy is essential for the treatment of lymphoma (Teske *et al*. 2002; Milner *et al*. 2005; Collette *et al*. 2016; Limmer *et al*. 2016). Reasons to consider surgical resection for intestinal lymphoma include gaining a histopathological diagnosis (Shales 2015), preventing or treating intestinal obstruction (MacPhail 2002) or perforation (Selting 2013). There is also a risk that a single discrete intestinal lymphoma may rupture with chemotherapy making surgery justifiable in these cases (Ettinger 2003). Recently, surgery followed by chemotherapy has been proposed as a treatment option for cats with discrete intermediate and high‐grade intestinal lymphomas (Gouldin *et al*. 2017). In humans with primary intestinal lymphoma, combined therapy with surgery and chemotherapy extends long‐term survival and has been recommended in most reports (Hong *et al*. 2017). Clearly surgery plays a role in the treatment of this disease, but there remains limited information in the veterinary literature as to the importance of this role. Further clarity can be provided through assessment of tumour extent within surgical margins of excised single discrete intestinal masses.

The aim of this study was to report the extent of tumour cell infiltration distant from the gross lesion in association with solitary neoplastic intestinal tumours in cats. The data generated will provide guidance relating to surgical and histopathological margins for specific intestinal tumour types in cats.

## Methods

This is a prospective, clinical study. Single visible and palpable discrete intestinal tumours removed surgically at both primary and referral veterinary centres across the United Kingdom and Ireland from March 2017 to March 2018 that were sent to Bridge Pathology Limited (www.bridgepathology.com) for histopathological assessment were collected post diagnosis for further investigation having been fixed in formalin. All small and large intestinal tumours presented in this period were included in the study if there was sufficient margin (a minimum of 1 cm of grossly normal intestinal tissue adjacent to the tumour) left for assessment after fixing and processing.

All masses were analysed further to determine the extent of tumour cells distant from the primary lesion in the excised sections, and to consequently determine tissue margins required to achieve complete resection in oral, aboral and mesenteric directions in each case. This analysis was performed in a similar manner to a previous veterinary study on canine cutaneous mast cell tumours (Simpson *et al*. 2004). A transverse sample was taken at the palpable tumour edge and then every 1 cm from this edge to the closest 1 cm increment to the surgeon‐cut tissue border. The surgeon‐cut tissue border for each mass was therefore always within 1 cm of the most distal centimetre in this study. The amount of tissue available for assessment varied from case to case. Because the aboral and oral directions were not known by the author (unless the mass was at the ileo‐caecal junction) one direction was termed the left side, the other the right side. Sampled tissue was then embedded in paraffin wax and cut at 4–5 microns before floating onto glass slides as previously described (Beck *et al*. 2011). These sections were routinely deparaffinised, rehydrated and stained with haematoxylin and eosin using a Gemini AS automated slide stainer (ThermoFisher Scientific, Cheshire, UK). The individual sections were examined by a single board certified pathologist (Sam Beck BSc BVSc MVetMed FRC‐Path MRCVS Dip.ACVP) to determine the presence or absence of neoplasia.

In addition to the results of the sectioned samples, the study used information obtained from the initial diagnostic histopathology reports. This included the diagnosis, evidence of metastatic spread to local lymph nodes (where available), and the presence or absence of neoplasia at the surgeon‐cut tissue border. Any discrepancies noted between the results of the examined sections in this study and the information in the initial histopathology reports were examined in more detail.

In this study, tumours were diagnosed either as carcinomas, sarcomas, mast cell tumours or lymphomas. The results of this study have been reported in a descriptive manner, and recommendations have been determined for histological margins in feline intestinal tumours for lymphomas and carcinomas individually, and for non‐lymphoma intestinal tumours collectively.

## Results

Over the 12‐month period, 32 intestinal tumours were diagnosed by Bridge Pathology Limited and deemed appropriate for this study. Two of these were subsequently excluded due to non‐neoplastic diagnoses. Of the remaining 30 tumours, 17 were intestinal lymphomas, nine were carcinomas, two were sarcomas and two were mast cell tumours.

For each neoplasm the intestinal tumour type, evidence for spread to local lymph nodes (where available), the left‐sided intestinal, right‐sided intestinal and mesenteric histological margins that would have resulted in complete tumour excision were recorded in Table 1. The initial diagnostic pathology reports for all cases are also included in Appendix S1.

**Table 1:** For each tumour the table includes the diagnosis and evidence for metastatic spread to adjacent lymph nodes

Case No	Histological diagnosis	Metastatic spread to local lymph nodes	First tissue section determined to be free of tumour cells, recorded as centimetres from the gross tumour margin on the left hand side	First tissue section determined to be free of tumour cells, recorded as centimetres from the gross tumour margin on the right hand side	First mesenteric section determined to be free of tumour cells, recorded as centimetres from the gross tumour margin
1	Intermediate grade lymphoma	Unknown	1 cm	0 cm	>2 cm
2	Intermediate grade lymphoma	Unknown	>4 cm	>1 cm	0 cm
6	Low grade lymphoma	Yes	>5 cm	>6 cm	n/a
7	High grade lymphoma	Unknown	1 cm	1 cm	1 cm
12	Intermediate grade lymphoma	Yes	7 cm	0 cm	>4 cm
14	High grade lymphoma	No	1 cm	0 cm	n/a
15	High grade lymphoma	Yes	6 cm	>1 cm	>0 cm
16	High grade lymphoma	Yes	2 cm	2 cm	1 cm
17	High grade lymphoma	Yes	>6 cm	>4 cm	>0 cm
19	Low grade lymphoma	Yes	5 cm	2 cm	>0 cm
21	Low grade lymphoma	Unknown	>1 cm	>1 cm	>1 cm
22	Intermediate grade lymphoma	Unknown	>2 cm	>6 cm	0 cm
25	Low grade lymphoma	Unknown	>1 cm	>2 cm	>1 cm
26	High grade lymphoma	Unknown	>3 cm	>0 cm	n/a
30	Intermediate grade lymphoma	Yes	n/a	0 cm	0 cm
31	Low grade lymphoma	No	1 cm	0 cm	0 cm
32	High grade lymphoma	Yes	2 cm	1 cm	0 cm
3	Carcinoma	Yes	4 cm	1 cm	n/a
4	Carcinoma	Yes	2 cm	1 cm	0 cm
5	Carcinoma	No	0 cm	2 cm	0 cm
11	Carcinoma	Unknown	3 cm	4 cm	Multiple mesenteric nodules noted
20	Carcinoma	Yes	2 cm	1 cm	0 cm
23	Carcinoma	No	1 cm	1 cm	0 cm
24	Carcinoma	Yes	0 cm	1 cm	1 cm
27	Carcinoma	Unknown	0 cm	0 cm	0 cm
28	Carcinoma	Unknown	0 cm	0 cm	>0 cm
9	Sarcoma	No	0 cm	1 cm	n/a
10	Sarcoma	No	>3 cm	2 cm	>1 cm
18	Mast cell tumour	Yes	3 cm	0 cm	0 cm
29	Mast cell tumour	Yes	5 cm	1 cm	n/a

The table also includes the first tissue section determined to be free of tumour cells, recorded as centimetres from the gross tumour margin on the left and right intestinal, as well as the mesenteric sides of each tumour. Please note, if there were neoplastic cells at the most distal section assessed then the result will appear as >‘X’ cm. For example, if the most distal segment assessed was 4 cm from the gross tumour and had neoplastic cell within the section then the result would appear as > 4 cm.

Seventeen of the 30 intestinal masses were small intestinal. Nine of these could be defined further as duodenal (2), jejunal (4) or ileal (3). Eleven were located at the level of the ileo‐caecal junction and two were colonic masses.

Twenty‐three of the cats in the study were noted to be domestic short‐haired cats, three were domestic long‐haired cats and there was one of each of the following breeds: Siamese, Norwegian forest cat, Persian and Bengal. The median age was 12 years (interquartile range (IQR) 5), with the median age for lymphoma diagnoses being 12 years (IQR 7) and those with carcinoma being 12 years and 6 months (IQR 4.5). The age of three cats in this study was not known. Thirteen of the cats in the study were male neutered, three were entire males, 12 were female neutered and two were entire female cats.

When assessing the 18 intestinal margins of the nine carcinomas, 6 of 18 margins were tumour free at 0 cm, 12 of 18 margins were tumour free at 1 cm, 15 of 18 were tumour free at 2 cm, 16 of 18 were tumour free at 3 cm and all 18 were tumour free at 4 cm. Therefore, when only considering the sections assessed in this study, all nine carcinomas would have been completely resected with 4 cm histological margins in oral and aboral directions. Of note, however, in one of the carcinoma cases (case 11) the initial diagnostic histopathology report showed neoplastic cells at the surgeon‐cut tissue borders. In the examined sections in this study the distal 1 cm in one direction and distal 1 cm and 2 cm in the other direction were free of neoplastic cells. The same pathologist assessed this carcinoma on both occasions. Also, in another of the carcinoma cases (case 28) our study showed a significant amount of margin clear of neoplastic cells in both directions (4 cm to the left hand side and 2 cm to the right hand side) while the initial diagnostic histopathology report showed neoplastic cells at both surgeon‐cut tissue borders. This case was assessed by a different pathologist on each occasion. Of the nine carcinomas in this study, only these two cases (cases 11 and 28) were observed to be histologically invading lymphatic vessels.

When assessing the histological mesenteric margins of the nine intestinal carcinomas, 5 of 9 margins were tumour free at 0 cm, and 6 of 9 margins were tumour free at 1 cm. Of the remaining three, one of the carcinomas was noted as having multiple mesenteric nodules, one mesenteric margin was not available for histologic assessment, and one carcinoma had neoplastic cells at the 0 cm section but no further mesentery was available for sectioning.

When assessing the four intestinal margins of the two sarcomas, one of four margins were tumour free at 0 cm, two of four margins were tumour free at 1 cm, three of four were tumour free at 2 cm, and all four margins were tumour free at 4 cm. Both sarcomas would have been completely removed with histological margins of 4 cm in oral and aboral directions. There was only one mesenteric margin available for assessment for the two sarcomas in the study. For this mesenteric margin, there were neoplastic cells present at the 1 cm section. No further mesentery was available for sectioning beyond the 1 cm measurement.

When assessing the four intestinal margins of the two mast cell tumours, one of four margins were tumour free at 0 cm, two of four margins were tumour free at 1 cm, three of four were tumour free at 3 cm and all four were tumour free at 5 cm. Both mast cell tumours would have been completely removed with histological margins of 5 cm. Only one mesenteric margin was available for assessment for the two intestinal mast cell tumours. This mesenteric margin was free of neoplastic cells at the 0 cm section.

Seven of 17 intestinal lymphomas were determined to be high‐grade, five were intermediate‐grade and five were low‐grade. All lymphomas were classified according to the World Health Organization (WHO) criteria (Valli *et al*. 2011). Only two of the 17 lymphomas in the study were immunophenotyped; one was a large high grade B‐cell colonic lymphoma, and the other an intermediate sized low grade T‐cell lymphoma of the duodenum.

Of the 17 intestinal lymphomas assessed, there was great variability in the histological margin required for complete resection of the discrete mass present. When assessing the 33 available intestinal margins for the 17 lymphomas, five of 33 margins were tumour free at 0 cm, 11 of 33 margins were tumour free at 1 cm, 15 of 33 were tumour free at 2 cm, 16 of 33 were tumour free at 5 cm, 17 off 33 were tumour free at 6 cm, and 18 of 33 were tumour free at 7 cm. Of the remaining 15 intestinal margins in the study, neoplastic cells were present in the most distal section available for assessment. In seven cases (cases 2, 15, 17, 19, 21, 22 and 26) there was a difference noted between the presence or absence of neoplastic cells at the surgeon‐cut tissue border in the initial diagnostic histopathology report and the distal 1 cm in our study. These two anatomic areas were within 1 cm of each other and in all cases had been assessed by a different pathologist. When assessing the 14 available histological mesenteric margins of the 17 intestinal lymphomas, five of 14 margins were tumour free at 0 cm, and seven of 14 margins were tumour free at 1 cm. Of the remaining seven mesenteric margins in the study, neoplastic cells were present in the most distal section available for assessment.

Neighbouring mesenteric lymph nodes were assessed histologically in 20 of the 30 cases in this study. Fourteen of these 20 cases had confirmed mesenteric lymph node metastasis. Six of nine carcinomas had the local lymph nodes biopsied at the time of surgery. Four of six had lymph node metastasis. Both of the sarcoma cases had their local lymph nodes biopsied at the time of surgery and in both cases the local lymph nodes were free of neoplastic infiltrate. Both mast cell tumours had their local lymph nodes biopsied at the time of surgery and in both cases there was lymph node metastasis. Ten of 17 lymphoma cases had local lymph node biopsied at the time of surgery. Of these 10 cases, eight had confirmed lymph node metastasis.

## Discussion

The results of this study show a clear difference between lymphoma and non‐lymphoma alimentary neoplasia in cats when assessing intestinal tumour histological margins. As a result the discussion in this article will focus on two areas. The first will be intestinal tumour histological margin recommendations for non‐lymphoma alimentary neoplasia. The second will be on the role of surgery for feline alimentary lymphoma.

Complete resection of intestinal tumours is important. In both humans and cats, survival time has been shown to be strongly influenced by the presence or absence of complete or incomplete surgical margins (Slawienski *et al*. 1997; Bakaeen *et al*. 2000; Green *et al*. 2011; Zhang *et al*. 2011)_._ In one human study of duodenal neoplasia, lymph node metastasis as well as positive resection margins had a significant negative impact on survival times in patients undergoing potentially curative surgery (Zhang *et al*. 2011). Another study showed that as long as a clean surgical margin can be secured there is no difference in survival times between those patients undergoing radical resection to those undergoing limited resection (Bakaeen *et al*. 2000). In the veterinary literature, there is a wide variety of recommendations when considering surgical margins for intestinal tumour removal in the cat (Table 2). In Table 2, a surgical margin with an upper limit of 8 cm is recommended in four of nine publications. Comparatively, the results of this study suggest clean surgical margins for non‐lymphoma single discrete intestinal neoplasia can be obtained with less tissue resection.

**Table 2:** Current recommendations for surgical margins when treating intestinal neoplasia in dogs and cats

Source	Intestinal surgical margin recommendation
(Marks 1996)	At least 4 cm for intestinal tumours in dogs and cats.
(Thamm 2015)	A generous margin for intestinal tumours in cats
(Marconato *et al*. 2013)	5–8 cm for both small and large intestinal tumours in cats
Tumours of the colon and rectum. In BSAVA Manual of Canine and Feline Oncology Third Edition (Bray 2011)	2–8 cm for colorectal neoplasia in dogs and cats.
Tumours of the gastrointestinal tract and associated structures. In Small Animal Oncology: An Introduction (North & Banks 2009)	4–8 cm for intestinal tumours in dogs and cats
BSAVA Manual of Canine and Feline Oncology Second Edition (White 2003)	Wide local resection with margins extending 4–8 cm for small intestinal tumours in dogs and cats.
(Seim 2003)	5–7 cm for intestinal adenocarcinoma in cats
Alimentary Tract. In Veterinary Surgical Oncology (Culp *et al*. 2012)	5 cm of small intestines for dogs and cats
Veterinary Surgery Small Animal Second Edition (Giuffrida & Cimino Brown 2018)	At least 3 cm of normal bowel and a similar amount of mesentery for small intestinal tumours in dogs and cats

The key consideration of this study is the measurement of margins in centimetres around the tumour when considering excision. Canine small intestine length will contract by 28.3% immediately after excision and by 26.3% after 24 h in formalin (Clarke *et al*. 2014). Human small intestine length will contract by 21.8% and the large intestine by 36.4% after 12 h in formalin (Wang *et al*. 2004). No similar feline study exists, and therefore in this study all margin recommendations are histological and not surgical. If feline intestinal shrinkage is similar to that of humans and dogs after formalin fixation then in Table 2 the surgical margin upper limit of 8 cm is likely to equate to a histological margin of approximately 6 cm. One human article discusses that the constitution and type of tissue may influence the degree of tissue shrinkage after formalin fixation. It also found that the average shrinkage of head and neck tumours after fixation was only 4.4%(Chen *et al*. 2012). As all the intestinal samples in this study contain neoplasia of varying types, sizes and level of infiltration it is likely that there is variability in the amount of tissue shrinkage between one segment and the next.

Seven of the nine carcinomas in this study would have been removed in their entirety with histological margins of 4 cm in oral and aboral directions. Whilst in this study the distal sections examined were free of neoplasia in all cases, in cases 11 and 28 the initial histopathology report confirmed the presence of neoplasia at both surgeon cut tissue borders. An explanation for this difference is that there was lymphovascular neoplastic invasion in both cases (please note that the other seven intestinal carcinomas in this study had no evidence for lymphovascular invasion). In the distal sections examined in this study neoplastic cells were not identified, and the tumour extended beyond the examined sections. In feline mammary carcinoma grading, lymphovascular invasion is associated with prognosis (Mills *et al*. 2015). The same may indeed be true for feline intestinal carcinomas. Based upon these limited case numbers, 4 cm histological margins would achieve complete excision of carcinomas in seven out of nine cases in oral and aboral directions. It is interesting to note that the two tumours that would not have been completely excised by a 4 cm excision also exhibited lymphovascular invasion. If these cases are truly representative of all intestinal carcinomas in cats, recommendations that a greater than 4 cm histological margin is taken would be unlikely to achieve a higher proportion of complete excisions due to lymphovascular invasion and trans‐serosal spread.

At the time of diagnosis intestinal carcinomas are rarely limited to the intestine (Marconato & Bettini 2013). In this study, five of the nine carcinomas metastasized to either local lymph nodes or other intra‐abdominal organs. This rate of metastatic spread is similar to that of previous reports (Patnaik *et al*. 1976; Green *et al*. 2011).

The two intestinal sarcomas in this study would have been fully excised with 4 cm histological margins. In both cases there was no evidence of metastasis to local mesenteric lymph node. Case 10 requires further explanation. In this study, tumour cells were present at the 3 cm segment on the left hand side. However, it was noted on the initial diagnostic histopathology report that both surgeon‐cut tissue borders were tumour‐free meaning a margin of 4 cm would have been sufficient for complete resection. As only two sarcomas were included in the study, little can be derived in way of margin recommendations for feline intestinal sarcomas.

Mast cell tumours are the third most common small intestinal neoplasia in cats (Shales 2015). Both mast cell tumours in this study would have been fully excised with 5 cm histological margins. Both, however, metastasized to the local mesenteric lymph nodes. Traditionally it has been thought that the biological behaviour of feline intestinal mast cell tumours is aggressive with a high metastatic potential and a low survival rate (Marconato & Bettini 2013). However, in a recent study of 31 cats with gastrointestinal mast cell tumours, the overall median survival time was 531 days, which suggests that the prognosis for cats with this disease may be better than previously reported (Barrett *et al*. 2018). In the 2018 study, surgery did not improve median survival time, meaning that complete resection may not be important in feline gastrointestinal mast cell tumours. Given that only two of these tumours are included in the study, little can be ascertained in the way of margin recommendations for feline intestinal mast cell tumours.

When assessing the seventeen lymphomas, there was a difference between the results of the surgeon‐cut tissue borders in the initial histopathology report and the reported distal segments in our series in seven cases. Cases 2, 15, 17, 21, 22 and 26 showed no neoplastic cells at the surgeon‐cut tissue borders, whilst in the distal centimetres assessed in our study there was evidence for neoplastic cells in either one or both sides. In case 19, the initial histopathology report showed neoplastic cells at one surgeon‐cut margin, but in both directions the distal 1 cm assessed in our series were free of neoplastic cells. All lymphomas in this study were overtly neoplastic and composed of atypical large round cells in unusual locations effacing pre‐existing architecture. Also, all were classified by WHO criteria (Valli *et al*. 2011) meaning it is unlikely that the differences discussed above were due to interpretative differences between pathologists. The variability is most likely explained by the segmental and patchy nature of lymphoma (Moore *et al*. 2012).

Feline lymphoma is considered a systemic disease (Ettinger 2003). The role of surgery in feline solitary discrete gastrointestinal lymphoma is not clear and is commonly restricted to patients with a probable obstruction, peritonitis and when a needle aspirate is not diagnostic (Gouldin *et al*. 2017). In the veterinary literature, it has previously been discussed that surgery followed by chemotherapy to treat intestinal lymphoma has not been demonstrated to improve survival compared with chemotherapy alone (Barrs & Beatty 2012; Selting 2013) and yet this recommendation is founded in only a few studies in which case numbers are small and the objective of the studies was not to assess this hypothesis (Mahony *et al*. 1995; Zwahlen *et al*. 1998).

In both feline and human medicine, there is evidence supporting the use of surgical resection alongside chemotherapy and other treatment modalities. In a recent study assessing 20 cats that had surgery followed by CHOP‐based chemotherapy for gastrointestinal lymphoma it was found that the disease free interval with such treatment was 357 days and the median survival time was 417 days (Gouldin *et al*. 2017). In humans, many authors have advocated a combination of surgery and chemotherapy to improve overall survival (Zinzani *et al*. 1997; Ibrahim *et al*. 2001; Kim *et al*. 2011; Gou *et al*. 2012; Hong *et al*. 2017). Surgical treatments are performed as the initial treatment followed by chemotherapy or radiation therapy if necessary. Yet the role of surgery in the treatment of humans with intestinal lymphoma remains controversial and unclear (Kobayashi *et al*. 2013; Abbott *et al*. 2015).

In this study, 17 of the 30 discrete intestinal tumours were lymphomas. It is not known why surgery was chosen in these 17 cases by the clinicians involved, be if for diagnosis or treatment. However, none of the tumours in this study were perforated. When the histological margins of these intestinal lymphomas were assessed, there is no ideal resection margin due to the variability in microscopic disease noted. In one human study, it was recommended that gross resection of the main lesion should be prioritized over achieving margin‐free status (Hong *et al*. 2017). Our study would support this. Importantly, it has been shown that there is not a high risk of post‐operative dehiscence after full thickness intestinal surgery in cats with lymphoma (Smith *et al*. 2011). If surgery is to be considered for either diagnosis or treatment of feline intestinal lymphoma then the surgical margin is unlikely to be important.

Diagnosis through ultrasound‐guided percutaneous fine needle aspiration of intestinal neoplasia is commonly accurate. It has a sensitivity of 71% and specificity of 100% when diagnosing intestinal lymphoma (Bonfanti *et al*. 2006). Histological assessment of intestinal biopsies is essential for the diagnosis of low‐grade feline intestinal lymphoma but is also required in diagnosis and immunophenotyping other forms of intestinal lymphoma where fine needle biopsy results are not diagnostic (Barrs & Beatty 2012). If the veterinary surgeon knows the type of tumour present prior to surgery then surgical margin planning can occur. Such planning will allow for better outcomes for the feline patient.

The biggest limitation of this study is the number of cases per tumour type. With only two of each of intestinal sarcomas and mast cell tumours little can be derived in way of surgical margin recommendations for these individual tumour types. Another weakness is the specific lack of knowledge about intestinal tissue shrinkage after formalin fixation in cats. Further research here would allow extrapolation of the results of this study from histological margin recommendations to surgical margin recommendations.

In conclusion, the outcome of the results of this study leads to the recommendation that discrete feline intestinal carcinomas should be removed with a margin in oral and aboral intestinal directions that would contract to 4 cm after fixation in formalin. This will lead to favourable surgical results unless there is evidence for lymphovascular invasion. Given the likelihood of spread to local lymph nodes and other abdominal organs with these tumour types, the local lymph nodes and any other abdominal abnormalities should be biopsied at the time of resection. If all the non‐lymphoma tumours evaluated in this study are considered together, then a surgical margin that would contract to 4 cm after formalin fixation in oral and aboral directions is most likely going to result in complete resection. The margins required when resecting single discrete feline intestinal lymphomas are less important. The aim should be to remove the gross tumour only. If possible, a diagnosis should be made prior to surgical resection in all cases to ensure good outcomes.

## Source of funding

None declared.

## Conflict of interest

None declared.

## Ethical statement

The authors confirm that the ethical policies of the journal, as noted on the journal's author guidelines page, have been adhered to. No ethical approval was required.

## Contributions

Deepthi Chandy and the staff at Bridge Pathology Ltd provided help in the collecting and processing of the tissue samples in this study.

## Supporting information


**Appendix S1.** Histopathology reports.
