# Supplementary material for: Evaluation of the histopathological extent of neoplastic infiltration in intestinal tumours in cats
Source: Vet Med Sci. 2019 Mar 11;5(3):307–16. doi: 10.1002/vms3.166 (PMC6682799; doi:10.1002/vms3.166)
Supplement: Supplementary file 1 — Appendix S1. Histopathology reports. [file VMS3-5-307-s001.docx]

Appendix 1- Histopathology reports

Case 1

**Clinical History:** Mass from small intestine. Appeared to have spread to mesenteric LN. Weight loss.

**Gross description:** A beige grey section of tubular tissue measuring 90x40x35mm, with a mass measuring 45x30mm. Representative sections taken.

**Histological description:** Small intestine (3 sections). The mucosa is extensively ulcerated and the submucosa and tunica muscularis are infiltrated by a non-encapsulated neoplasm. Neoplastic cells are arranged in sheets supported by a fibrovascular stroma. Neoplastic cells are round with a moderate amount of eosinophilic cytoplasm and indistinct cell borders. Nuclei are oval, measure approximately 2-2.5x the diameter of adjacent erythrocytes, with finely stippled chromatin and 1 magenta nucleolus. There is moderate anisocytosis and anisokaryosis. Mitoses average 6-7 per high power field. Neoplastic cells multifocally infiltrate the adjacent mesentery. There are multiple foci of necrosis. Within sections through the proximal and distal margin of the submitted segment the mucosal architecture is overall preserved. Within one of the sections the lamina propria is infiltrated by moderately increased lymphocytes and plasma cells. Lymphocytes are of small size. There are occasional aggregates of neutrophils and eosinophilic material within the crypt lumen.

**Histological diagnosis:** Round cell tumour, consistent with lymphoma, small intestine.

**Comment:** The mass within the small intestine is a round cell tumour. The histological features are suggestive of lymphoma and other round cell tumours, such as a mast cell tumour, are considered much less likely. Immunohistochemistry can be performed on this sample on request for further characterisation of the neoplastic population (lymphoma typing). The mass extends from the intestine into the adjacent mesentery. No obvious neoplastic tissue is observed within the sections through the surgical borders of this segment. Within one of these sections there are increased numbers of lymphocytes, however these are small and are admixed with plasma cells and neutrophils. Therefore these are interpreted to represent an inflammatory population. Therefore the mass appears to have been completely excised but there is potential for metastasis to lymph nodes and distant sites.

Case 2

**Clinical History:** GA laparotomy for abdominal mass. PAB profile ALP - 55(10-90) ALT - 34 (20-100) BUN - 5.6 (3.6-10.7) CRE - 97 (3.9-8.3) GLU - 9.8 (3.9-8.3)

**Gross description:** A beige section of tubular tissue measuring 75x40x25mm with a firm white mass measuring 30x30mm. Representative sections taken.

**Histological description:** Small intestine (4 sections). The intestinal architecture is focally extensively effaced and the mucosa, submucosa and tunica muscularis are infiltrated by a poorly demarcated neoplasm. Neoplastic cells are arranged in sheets supported by a fine fibrovascular stroma. Neoplastic cells are round, with a moderate amount of eosinophilic cytoplasm and indistinct cell borders. Nuclei are oval, measure approximately 2.5 times the diameter of adjacent erythrocytes, with finely clumped chromatin and one nucleolus. There is moderate anisocytosis and anisokaryosis. Mitoses average 7-8 per high power field. The surface of the mass is extensively ulcerated and large aggregates of fibrin and degenerate neutrophils are present on the surface. These are admixed with large colonies of bacteria. Within sections through the proximal and distal

surgeon-cut tissue borders of the submitted segment the intestinal architecture is preserved. Intestinal villi are long and slender. Numbers of lymphocytes and plasma cells within the lamina propria are mildly increased. Lymphocytes in these areas are of small size.

**Histological diagnosis:** Consistent with lymphoma, small intestine.

**Comment:** The mass within the small intestine is a round cell tumour and the features are consistent with lymphoma. Further characterisation of the neoplastic population using immunohistochemistry can be performed on this sample on request (lymphoma typing). No obvious neoplastic tissue is

observed within the sections through the proximal and distal margins of the submitted segment. Therefore full excision appears to have been achieved. Nevertheless, this tumour is expected to have potential for metastasis from this site.

Case 3

**Clinical History:**

1. Ileal caecal junction and colon resection. 2. Pancreatic cyst - histo. Previous FNA - neutrophilic infiltration of colon. Focaul mass ileocaecal junction and second mass at colon with associated enlarged lymph nodes. Pancreatic cyst - incidental finding on abdo ultrasound. Adenocarcinoma vs lymphoma vs granuloma. LN mets?

**Gross description:**

A: Ileal caecal junction + colon. A beige-brown section of tubular tissue measuring 110x35x20mm. Representative sections taken.

B: Pancreatic cyst. 2 beige brown irregular wedges of tissue measuring up to 30x20x15mm. Representative sections taken.

**Histological description:**

A. Ileocolic junction (5 sections). Multiple transverse sections of tissue from both the ileocaecal junction as well as a discrete region of the colon, show a markedly infiltrative, poorly demarcated, transmural neoplastic mass that has been fully excised with the resected portion of intestines. The mass is composed of irregular tubular structures supported by an abundant fibrous to desmoplastic stroma. The neoplastic tubules are typically one layer thick and are often

dilated and filled with necrotic cellular debris and degenerate neutrophils. Individual neoplastic cells are polygonal to columnar with indistinct cell borders and a moderate amount of eosinophilic cytoplasm. Nuclei are typically oval with stippled to clumped chromatin and often have a single prominent nucleolus. There is moderate anisocytosis and anisokaryosis, and there are up to 4 mitotic figures in a single high power field. Throughout the neoplastic population there are also numerous goblet-type cells. The neoplastic tubules arise in the mucosa but extends through the submucosa and muscularis out to the serosa. Many of the tubules are filled with a moderate to abundant amount of streaming, pale amphophilic mucoid material. Admixed throughout the supporting stroma is a moderate, mixed inflammatory infiltrate, variably composed of lymphocytes and plasma cells in some regions and of neutrophils and macrophages in other regions. Towards the periphery of the mass, there are regions of haemorrhage and associated aggregates of haemosiderin-laden macrophages. There are rare

multinucleated giant cells associated with areas of inflammation and haemorrhage. Transverse sections of tissue from the ileal and colonic margins show no evidence of the neoplastic cells.

B. Pancreatic cyst (4 sections). The examined sections of tissue are largely composed of thick tracts of smooth muscle that merge into fibrous stroma. Some sections are lined along one edge by a single layer of tall columnar epithelial cells and occasionally the opposite edge is lined by a border of variably loose to dense fibrovascular stroma. Rarely, there are small aggregates of lymphocytes and plasma cells present in a perivascular pattern.

**Histological diagnosis:**

A. Carcinoma; with enteritis, ulcerative, neutrophilic, lymphocytic, and plasmacytic, ileocaecal junction and colon.

B. Consistent with endodermal cyst, tissue described as pancreatic cyst.

**Comment:** As suspected, there is a neoplastic mass present at the ileocaecal junction as well as a second region within colon. Histology is consistent with an adenocarcinoma with frequent mucinous type differentiation. There is marked inflammation throughout this mass, consistent with the previous cytology findings of neutrophilic inflammation. The history indicates the involvement of

lymph nodes, and there is a small mass-like structure, immediately adjacent to the ileocaecal junction that is composed entirely of neoplastic and inflamed tissue. It is possible that this represents a lymph node that has been entirely effaced by the neoplasm, however there is no remaining tissue to confirm origin as a lymph node. This neoplasm is expected to be both locally infiltrative and does have a potential for metastasis and/or carcinomatosis. The tissue submitted from the described pancreatic cyst is composed primarily of smooth muscle with smaller amount of fibrous stroma and a single layer of epithelial lining. These features including the thick layer of smooth muscle is most suggestive of a congenital endodermal cyst. There is no evidence of a neoplastic population within this tissue, and excision is likely to be curative.

Case 4

**Clinical History:** Ileocaecal junction mass with mesenteric lymphadenopathy. Clinical info: weight loss, slightly high WBC.

**Gross description:** Intestine- A beige section of tubular tissue measuring 65x32x20mm. Representative sections taken.

**Histological description:** Ileocaecal junction (5 sections). Sections which are representative of the expanded area include a piece of compressed small intestine at one side of the transverse sections with moderately hyperplastic lymphoid nodules associated with the mucosa. Within the submucosa of this area there are widely patent lymphatics. This area of recognisable intestine abuts an area of irregular fibrous proliferation within which there is a necrotic tract. Within the fibrous proliferation are multiple scattered variably-sized cell nests and acinar structures. These nests and acini are lined by cuboidal to columnar epithelial cells with indistinct cell margins and modest amounts of palely eosinophilic cytoplasm. Nuclei are round to oval with coarsely stippled chromatin and scattered mitoses are observed within these cells. There is multifocal necrosis with accumulations of mucus and also neutrophils. Within two areas of lymphoid tissue (lymph node) which protrude from the outer surface of this irregular section and neoplastic acinar structures are observed within the lymphoid parenchyma. Sections from the excision edges of the specimen have widely patent lymphatics and lacteals (oedema) but do not include neoplastic cells.

**Histological diagnosis:** Adenocarcinoma, with lymphoid metastases, ileocaecal junction.

**Comment:** The histological appearance of the described mass at the ileocaecal junction is consistent with presence of an adenocarcinoma. No neoplastic cells are observed within the excision margins of the specimen however spread of

the neoplastic cells into lymph nodes in the edges of the tumour is recognised and therefore more distant spread cannot be ruled out.

Case 5

**Clinical History:** Enlargement / thickening of distal ileum / caecum colon ex-lap performed. Mass removal ilio-caecal junction.

**Gross description:** A beige grey section of tubular tissue measuring 65x35x20mm, with a mass measuring 30x35mm. Representative sections taken.

**Histological description:** Ileocaecocolic junction (4 sections, 2 levels including abaxial and aboral margins). Expanding the mucosal surface, extending through the subjacent muscularis mucosa into the submucosa and inner muscular layers is a poorly demarcated, unencapsulated, infiltrative, epithelial neoplasm arranged in acini, nests and small packets, supported by a moderate fibrous stroma (desmoplasia). Neoplastic cells are large, polygonal, moderately pleomorphic, with variably distinct cell borders, moderate eosinophilic cytoplasm with a single, oval to irregular oval nucleus, which contains a prominent nucleolus and stippled chromatin. There is moderate to

marked anisocytosis and anisokaryosis, with 18 mitoses observed within the three high power fields available for examination. Neoplastic cells are multifocally replaced by eosinophilic cellular and karyorrhectic debris (necrosis), with separation and infiltration by moderate to high numbers

of neutrophils, lymphocytes, plasma cells and macrophages. Neoplastic acini frequently contain accumulations of necrotic debris. Segmentally, the mucosal surface is ulcerated, replaced by a coagulum composed of necrotic debris and moderate to high numbers of viable and degenerate neutrophils admixed with high numbers of bacterial colonies of mixed morphology. The surrounding propria is expanded by proliferation by a fibrous stroma, which extends into the subjacent submucosa and muscular layers. Further bordering these areas there are dense clusters of lymphocytes and plasma cells, which are frequently present in a perivascular location. Neoplastic cells are not present at the oral and aboral margins.

**Histological diagnosis:** Adenocarcinoma, ileocaecocolic junction.

**Comment:** The mass at the ileocaecocolic junction represents an adenocarcinoma, which has likely arisen from the cryptal epithelium. The neoplasm appears moderately infiltrative, with effacement and ulceration of the mucosa, initiating a moderate to marked inflammatory response. These

tumours may metastasise and therefore monitoring of draining lymph nodes/further sites such as the lung fields may be considered in this case. Neoplastic cells are not observed at the oral and aboral margins.

Case 6

**Clinical History:** Presented for treatment of intussusception/mass. Prev history of EPI, hypocobalaminemia. Inappetent for 3 weeks, straining to defecate for 1 week, nausea, anorexia for 1 week.

**Gross description:**

A: Intestinal mass resection. A beige grey section of tubular tissue measuring 160x15x8mm with a sessile mass measuring 6x6mm. Representative sections taken.

B: Mesenteric lymph node. 2 beige brown irregular wedges of tissue measuring up to 6x3x2mm. Embedded whole. All tissue used.

**Histological description:**

A. Intestinal mass (9 sections). Infiltrating and expanding the lamina propria throughout the sections and extending to the end of the intestinal segment, and infiltrating into the underlying submucosa and muscularis layers, is a densely cellular neoplastic mass. Neoplastic cells are arranged in sheets and in cords, supported by the pre-existing stroma. Individual neoplastic cells are round to polygonal and contain scant eosinophilic cytoplasm with variably distinct cell

borders. Nuclei are oval to round and measure approximately the same diameter as adjacent erythrocytes. Nuclei contain densely stippled to clumped chromatin. Mitoses are less than 1 per high power field. Extensive infiltration into the mucosal epithelium of the villi by the neoplastic cells is not apparent.

B. Mesenteric lymph node (2 sections). The lymph node parenchyma throughout the sections is partially disrupted by densely packed sheets of neoplastic cells with the same features as those described above.

**Histological diagnosis:** Lymphoma, small cell, low grade, small intestine and mesenteric lymph node.

**Comment:** There is a population of atypical small lymphocytes infiltrating throughout the mucosa of the intestinal segment which extend to the tissue ends, and into the underlying submucosa and muscularis layers. These features are indicative of an indolent to low grade lymphoma type, which has also partly extended to the associated lymph node. There is no evidence of epitheliotropism, suggesting that this is not an epitheliotropic T cell lymphoma, however it may represent an intestinal type T-cell lymphoma or lymphoma of B cell origin. Immunohistochemistry would be required to subclassify the lymphoma further, according to the WHO lymphoma classification, although this may not affect the prognosis. The very low mitotic activity of the neoplastic cells together with the small size of the nuclei indicate an indolent to low grade, slowly progressive lymphoma type.

Case 7

**Clinical History:** Jejunal mass. See submission form.

**Gross description:** Jejunum**-** A grey section of tubular tissue measuring 200x30x20mm, with a mass measuring 30x20mm. Representative sections taken.

**Histological description:** Jejunum (4 sections). Multifocally the intestinal mucosa, submucosa, tunica muscularis and adjacent mesentery, are infiltrated by a non-encapsulated neoplasm. Neoplastic cells are arranged in sheets, supported by a fine fibrovascular stroma. Neoplastic cells are round with a moderate amount of pale eosinophilic cytoplasm and indistinct cell borders. Nuclei are oval, measure approximately 2.5x the diameter of adjacent erythrocytes, with finely stippled chromatin and 1 large magenta nucleolus. There is moderate anisocytosis and anisokaryosis. Mitoses average 10 per high power field. Low numbers of small and intermediate size lymphocytes are present. The mucosa is multifocally extensively ulcerated and large aggregates of fibrin and

neutrophils are present on the ulcerated surface. Within sections through the surgeon-cut tissue borders of the submitted intestinal segment, no obvious neoplastic population is identified.

**Histological diagnosis:** Consistent with lymphoma, large cell, high grade, jejunum.

**Comment:** The mass within the jejunum is composed of round cells which are predominantly of large size. A plasmacytoid appearance (as seen in the cytology samples of the spleen and liver) is not obvious and the histological features support the cytology results for the jejunum. The mitotic count is consistent with a high grade lymphoma. Further characterisation of the neoplastic population using immunohistochemistry can be performed on request (lymphoma typing). No obvious neoplastic tissue is identified within the surgeon-cut tissue borders of the submitted intestinal segment. However there is likely potential for metastasis from this site.

Case 9

**Clinical History:** Intestinal mass distal jejunum on mesenteric border excise with margins. Also bx mesenteric lymph node.

**Gross description:**

A: Lymph node biopsy. A beige irregular piece of tissue measuring 5x3x3mm. Embedded whole. All tissue used.

B: Mass distal duodenum. A beige grey section of tubular tissue measuring 70x35x25mm with a mass measuring 20x35mm. Representative sections taken.

**Histological description:**

A. Mesenteric lymph node (1 section). The lymph node parenchyma throughout the section is very well organised. Small numbers of well organised follicles are present within the cortex. Moderate accumulations of small lymphocytes are present within the paracortex. Small numbers of macrophages and lymphocytes are present within sinuses.

B. Distal jejunum (4 sections). Within the muscularis layers and extending into the adjacent serosa at the site of the mass identified grossly, is a poorly demarcated, locally infiltrative, densely cellular, nonencapsulated neoplastic mass. Neoplastic cells are arranged in moderately densely packed, interlacing short streams, supported by small amounts of collagenous stroma. There is extensive cavitation with accumulations of erythrocytes and necrotic cellular debris (comprising up to 50% of the total neoplastic cell area in some sections) within the mass. Individual neoplastic cells are spindle-shaped, contain moderate amounts of eosinophilic cytoplasm and have indistinct cell borders. Nuclei are elongated oval and contained lightly stippled chromatin with 1-2 variably prominent nucleoli. Mitoses are 2 per 10 high-power fields. No neoplastic cells are apparent at the proximal or distal surgeon-cut edges of the tissue segment.

**Histological diagnosis:**

A. Lymphoid hyperplasia, mild, mesenteric lymph node.

B. Soft tissue sarcoma, distal duodenumn.

**Comment:**

This mass from the duodenum represents a sarcoma, for which the differential diagnosis is a leiomyosarcoma or a gastrointestinal stromal tumour (GIST). Immunohistochemistry (4 antibodies) would be required to distinguish between these different tumour types and indeed this may be prognostically relevant given that GISTs would be expected to express CD117 (kit) and therefore maybe amendable to therapy with the new generation of receptor tyrosine kinase

inhibitors. The mass has been fully excised with no neoplastic cells at the proximal or distal surgeon-cut edges of the tissue and no neoplastic cells are present within the separately submitted lymph node.

Case 10

**Clinical History:** 2 samples. 1- from mesenteric lymph node small punch biopsy. 2- from small intestine, nodular mass on mesenteric side of intestine. Suspect neoplasia.

**Gross description:**

A: A beige grey irregular piece of tissue measuring 3x3x3mm. Embedded whole. All tissue used.

B: A beige section of tubular tissue measuring 65x25x20mm. Representative sections taken.

**Histological description:**

a. lymph node (1 section – 4 levels). The lymph node parenchyma throughout the tissue is well organised with densely packed small to intermediate size lymphocytes present. There are moderate accumulations of well organised hypertrophied endothelial cells. No neoplastic cells are apparent.

b. Small intestine (4 sections). At the site of the mass identified grossly, is a locally infiltrative, densely cellular neoplastic mass that extends through the muscularis layers and to the ulcerated mucosal surface. The mass is composed of intersecting bundles and streams of neoplastic cells that are supported by moderate amounts of collagenous matrix. Individual neoplastic cells are

spindle-shaped to occasionally polygonal and contain abundant eosinophilic cytoplasm with indistinct cell borders. Nuclei are large, oval to irregular oval and contain stippled to dispersed chromatin with 1-3 prominent, often large nucleoli. There is a marked degree of anisokaryosis and anisocytosis throughout the neoplastic cell population. With scattered multinucleated cells present. Mitoses are 22 per 10 high power fields. Extensive areas of confluent necrosis are present within the mass, representing up to 40% of the total mass area. Large numbers of small lymphocytes and scattered clusters of neutrophils and eosinophils are infiltrating amongst this neoplastic cell population. Extensive accumulations of necrotic cellular debris and bacterial organisms are present ion the ulcerated mucosal surface. Neoplastic cells are not apparent at the proximal or distal surgeon-cut edges of the intestinal segment. Within the distal and proximal ends of the intestinal segment, mucosal villi are moderately tall and are lined by tall columnar epithelial cells with moderate numbers of goblet cells present. Intraepithelial lymphocytes are present at a frequency of up to 30 cells per high power field stretch. Increased numbers of small lymphocytes and plasma cells are present throughout the supporting laminar propria representing approximately 60% of the total tissue areas with 1-4 cells per intercrypt space. Eosinophils amongst tis cell population are present at a frequency of up to 10 cells per high power field. Multiple well organised dense lymphoid follicular aggregates are present within some areas.

**Histological diagnosis:**

a. No significant lesions, mesenteric lymph node.

b. Sarcoma, poorly differentiated, with enteritis, ulcerative, chronic, segmental, marked and enteritis, lymphocytic, plasmacytic and eosinophilic, chronic, moderate, small intestine.

**Comment:**

The mass within the intestinal segment represents a sarcoma, most likely of fibroblast or smooth muscle origin, with the main differential diagnosis being a gastrointestinal stromal tumour (GIST). The mass does appear to have been fully excised, but given the high grade histological features, there would be significant potential for metastasis. Should metastasis occur, it may develop within the lungs, or in other abdominal organs such as the liver. No neoplastic cells are apparent within the submitted sample of mesenteric lymph node. There are also chronic inflammatory changes within the intestinal segment which extend to the tissue ends which may reflect an ongoing chronic hypersensitivity-type process which is likely separate from the tumour itself.

Case 11

**Clinical History:** Chronic inappetence/weight loss/vomiting. Ex lap after FNAS non-diagnostic. Liver diffusely abnormal - palpable nodules. Spleen focal white nodules. Ileocaecocolic junction - mural mass lesion and diffuse lymphadenopathy. Omentum scattered nodules.

**Gross description:**

A- Spleen. A brown irregular wedge of tissue measuring 15x11x6mm, with a mass measuring 3x3mm. Trisected. All tissue used.

B- Liver. A brown irregular wedge of tissue measuring 18x10x8mm. Bisected. All tissue used.

C- Omentum. A beige-brown irregular piece of tissue measuring 55x20x3mm. Representative sections taken.

D- Ileoceaco colic junction. A beige section of tubular tissue measuring 110x40x22mm. Representative sections taken.

**Histological description:**

A. Spleen (3 sections, 2 levels). Expanding and replacing up to 50% of each section, is a densely cellular, unencapsulated, moderately infiltrative neoplasm forming sheets, nests and lobules, supported by pre-existing stroma. Neoplastic cells are large, polygonal, with variably distinct cell borders, abundant eosinophilic, granular cytoplasm that contains a single irregular oval nucleus, with a large nucleolus and stippled chromatin. There is moderate anisokaryosis

and anisocytosis, with 32 mitoses observed per high power field available for examination. Neoplastic cells are frequently bordered by high numbers of neutrophil and extend to within 1mm of the surgeon-cut tissue borders.

B. Liver (2 sections, 2 levels). Multifocally and randomly, up to 50% of each section is replaced by the neoplastic population as described above arranged in nests and packets supported by the pre-existing stroma. Neoplastic cells are large, polygonal, with variably distinct cell borders, abundant eosinophilic, granular cytoplasm that contains a single irregular oval nucleus, with a

large nucleolus and stippled chromatin. There is moderate anisokaryosis and anisocytosis, with 44 mitoses observed per high power field available for examination. Neoplastic cells are present at the surgeon-cut tissue borders.

C. Omentum (3 sections). Multifocally and randomly, up to 80% of each section is replaced by the neoplastic population as described above, forming isolated nests and cords, supported by dense fibrous stroma (desmoplasia). Neoplastic cells are large, polygonal, with variably distinct cell borders, abundant eosinophilic, granular cytoplasm that contains a single irregular oval nucleus, with a large nucleolus and stippled chromatin. There is moderate anisokaryosis and

anisocytosis, with 50 mitoses observed in ten (x400) high power fields. Frequently neoplastic cells are bordered by low to moderate numbers of plasma cells, lymphocytes and fewer neutrophils. Mesothelial cells are frequently apically rounded and plump (activated). Neoplastic cells are present at the surgeon-cut tissue borders.

D. Ileocaecocolic junction (5 sections). Extending from, and subtotally replacing the mucosa,

infiltrating and replacing the submucosa, muscular layers, serosa and mesentery is a highly infiltrative, unencapsulated, densely cellular epithelium neoplasm arranged in nests, acini and lobules, supported by moderate to dense fibrous stroma (desmoplasia). Neoplastic cells are large, polygonal, with variably distinct cell borders , moderate to abundant eosinophilic cytoplasm that contains a single oval nucleus, with a large nucleolus and stippled chromatin. There is moderate anisokaryosis and anisocytosis and 24 mitoses are noted in a single high power field. Neoplastic cells are frequently individually necrotic and acini contain sloughed neoplastic cells admixed with necrotic debris. Coalescing areas of the neoplasm are necrotic and infiltrated and bordered by a mixed inflammatory population including moderate to high numbers of neutrophils, lymphocytes and plasma cells. Large numbers of neoplastic cells are observed within lymphatics

forming discrete emboli. Neoplastic cells are present at the surgeon-cut tissue borders.

**Histological diagnosis:**

A. Adenocarcinoma, metastatic; spleen.

B. Adenocarcinoma, metastatic; liver.

C. Adenocarcinoma, metastatic; omentum.

D. Adenocarcinoma; ileocaecocolic junction.

**Comment:**

Examination of the ileocaecocolic junction reveals the presence of a highly infiltrative adenocarcinoma that has most likely formed following neoplastic transformation of the cryptal epithelium. The neoplastic population has invaded the mucosa, transmurally infiltrated to the serosa and is present within vessels. This is consistent with the presence of a similar population within the spleen and randomly distributed throughout the liver. As well as the presence of

distant lymphovascular metastasis there is also evidence of transcoelomic metastasis in the omentum. The population at these distant sites has a markedly high mitotic index and an aggressive clinical course is suspected. Further distant metastasis and local progression are expected in this case.

Case 12

**Clinical History:** Ileal mass, caecum, ileo caecocolic junction. Ileal mass with loss of wall layering on ultrasound, resection of mass, caecum and ileocaecalcolic junction. Suspected lymphoma. Cytology sample non diagnostic.

**Gross description:** Ileum. A beige grey section of tubular tissue measuring 370x40x25m. Representative sections taken.

**Histological description:** Ileocaecocolic junction (5 sections). Segmentally expanding the wall of the ileum is a neoplastic mass that extends transmurally from the mucosa through the submucosa and muscularis out to the serosa. The mass is composed of densely cellular sheets of neoplastic cells supported by a

scant to moderate amount of pre-existing and reactive fibrovascular stroma. The tissue is relatively poorly fixed and cellular detail is somewhat obscured, however, the individual neoplastic cells appear round to polygonal with variably discrete cell borders and a scant amount of eosinophilic cytoplasm. Nuclei are oval and variably contain stippled to clumped chromatin and occasionally a single large prominent nucleolus. Nuclei are sometimes greater than 2 red

cells in diameter. Throughout the mass there is moderate anisocytosis and anisokaryosis, and in many regions there are 5-10 mitotic figures per single high power fields. The neoplastic cells are admixed with abundant inflammatory cells including plasma cells, neutrophils and occasional macrophages. Multifocally throughout the mass there are small foci of necrosis with more intense aggregates of neutrophils and macrophages surrounding these regions. The mucosa is entirely ulcerated and replaced by a band of necrotic material with frequent bacterial colonies. In the surrounding mesentery, some lymph nodes are expanded by the same neoplastic population. In other lymph nodes, there is a mixed inflammatory infiltrate, largely composed of neutrophils and macrophages with increased numbers of plasma cells. Transverse sections of tissue from the orad and aborad margins of the intestine are examined

with no evidence of neoplastic cells present.

**Histological diagnosis:** Compatible with lymphoma, large cell, intermediate grade, transmural, with lymphadenitis, pyogranulomatous, ileum and ileocaecocolic lymph nodes.

**Comment:** Consistent with the clinical suspicion, the histologic findings are most suggestive of a lymphoma. There is a transmural infiltrate throughout the ileum and there are fields which are quite suggestive of lymphoma. However, the tissue is somewhat poorly fixed which slightly obscures cellular detail and in addition, there is a prominent inflammatory infiltrate throughout much of the tissue that also somewhat obscures the neoplastic population. This same mix of a neoplastic

population as well as mixed inflammation is seen in the surrounding lymph nodes at the ileocaecalcolic junction. Additional immunohistochemistry would be suggested to confirm this diagnosis and determine the immunophenotype, assuming this is a lymphoma. Please call if you would like to request this additional diagnostic test.

Case 14

**Clinical History:** Intestinal mass and oment. Chronic weight loss, polyuria/polydipsia/polyphagia. Large abdominal mass (involving the cecocolic junction). Please check if margins are complete.

**Gross description:**

A- A beige grey section of tubular tissue measuring 170x55x40mm. Representative sections taken.

B- Omentum. A beige sheet of tissue measuring 70x50x3mm. Representative sections taken.

**Histological description:**

A. Cecocolic junction (6 sections). Infiltrating from the ulcerated mucosal surface and throughout all layers and to the subserosal stroma, is a densely cellular, non encapsulated neoplastic mass. Neoplastic cells are arranged in dense sheets, supported by small amounts of fibrovascular connective tissue stroma. Individual neoplastic cells are round, contain small amounts of eosinophilic cytoplasm and have variably distinct cell borders. Nuclei are round to oval and

contain stippled to clumped chromatin with 1-3 small nucleoli. Mitoses are up to 10 per high power field. Nuclei measure approximately 1-1.5x the diameter of adjacent erythrocytes. Tingible body macrophages are present at a frequency of up to 20 cells per high power field. Neoplastic cells are not apparent at the distal or proximal surgeon-cut ends of the intestinal segment. Occasional well organised lymphoid follicular structures are present within the submucosa at

these distal and proximal surgeon-cut ends.

B. Omentum (multiple sections). Neoplastic cells with the same features as those described above are widely infiltrating throughout the tissue.

**Histological diagnosis:** A and B. Lymphoma, high grade, intermediate size, diffuse, cecocolic junction with metastasis, omentum.

**Comment:** The abdominal mass represents a high grade lymphoma which has metastasised to the omentum. The neoplastic cells do not extend to the proximal or distal surgeon-cut ends of the intestinal segment, but given the histological findings and the high grade nature of the lymphoma, surgical excision is still unlikely to prove curative as this is part of a systemic disease. Further extension to other intestinal sites and to other internal organs is likely to develop with

time. Immunohistochemistry would be required to subclassify the lymphoma further according to the WHO lymphoma classification scheme.

Case 15

**Clinical History:** Mass (previous FNAs suggestive of lymphoma) distal ileum, proximal to ileo/caecal junction. Excised, margins taken aborally, very limited adorally. Multiple large lymph nodes removed.

**Gross description:**

a- 8 beige brown irregular pieces of tissue measuring up to 45x12x7mm. Representative sections taken.

b- A beige grey section of tubular tissue measuring 90x25x30mm. Representative sections taken.

**Histological description:**

a. Intestinal lymph nodes (8 sections). These sections represent intestinal lymph nodes. In all of these lymph nodes, the cortex is expanded by multiple, variably sized and well organised polar secondary lymphoid follicles. Medullary cords are variably expanded by abundant plasma cells and rare admixed Mott cells. In some of these sections, medullary sinuses are markedly expanded (oedema). In one of the sections 9from the largest lymph node), the cortical and focally also the subcapsular sinus is expanded by a population of round cells, which is very

similar to the population of neoplastic cells described in sample ‘b’. These are focally admixed with moderate numbers of eosinophils.

b. Distal ileum (4 sections). Effacing the mucosa in a focally extensive area and infiltrating the submucosa and muscular wall, is a dense neoplastic round cell infiltrate. Neoplastic cells are round to polygonal with indistinct cell borders and contain moderate amounts of pale eosinophilic cytoplasm. Nuclei are irregular round to oval and occasionally indented with coarsely stippled to clumped chromatin and often a single, medium sized distinct magenta nucleolus. Mitoses are up to 13 per high power field. Nuclei are up to 3x the size of adjacent erythrocytes. Multifocal small areas of necrosis are present. Neoplastic cells frequently infiltrate to within 1 mm of the serosal lining. In sections from the proximal and distal margin, no obvious infiltration with neoplastic cells is evident. In one of the sections, Peyer’s patches are hyperplastic but form distinct well differentiated secondary lymphoid follicles.

**Histological diagnosis:**

a. Lymph node metastasis of lymphoma, large cell, high grade, mesenteric lymph nodes.

b. Lymphoma, large cell, high grade, distal ileum.

**Comment:** Consistent with the FNA results, the submitted mass from the distal ileum represents a lymphoma. Neoplastic cells are large with a high mitotic activity, hence this lymphoma has been scored a high grade. Complete local excision seems to have been achieved, however metastasis to one of the submitted lymph nodes (the largest) has occurred. This tumour would be expected to have potential for further spread within the alimentary tract, regional lymph nodes and distant sites.

Case 16

**Clinical History:** Intestinal mass (descending colon). Enlarged mesenteric lymph nodes suspicious of lymphoma.

**Macroscopic Description:**
A- A beige grey section of tubular tissue measuring 80x35x30mm. Representative sections taken. 
B- LN. A beige piece of ovoid shaped tissue measuring 20x10x4mm. Bisected. All tissue used.
**Microscopic Description:**
A.  Descending colon (4 sections).   The colonic architecture is focally extensively effaced and the mucosa, submucosa and tunica muscularis are infiltrated by a non-encapsulated neoplasm.  Neoplastic cells are arranged in sheets, supported by a fine fibrovascular stroma.  Neoplastic cells are round with a moderate amount of eosinophilic cytoplasm and indistinct cell borders.  Nuclei are oval, measure approximately 2.5 - 3 x the diameter of adjacent erythrocytes, with finely clumped chromatin and one, often large magenta nucleolus.  There is moderate anisocytosis and anisokaryosis.  Mitoses average 9-10 per high power field.  Neoplastic cells multifocally extend into the adjacent mesentery.  No obvious neoplastic tissue is observed within sections through the colon at the surgeon-cut tissue borders.  
A & B.  Mesenteric lymph nodes (4 sections).   Within the mesenteric lymph nodes included with the colonic segment, as well as in the lymph nodes submitted separately, the cortex is multifocally effaced by aggregates of neoplastic cells, which resemble those described for the colonic wall above.
**Immunohistochemical Description:**
Neoplastic cells exhibit cytoplasmic and membranous labelling for CD20 and nuclear labelling for Pax-5. Scattered lymphocytes exhibiting cytoplasmic and membranous labelling for CD3 are present within the stroma.
**Final Diagnosis:**
A. B-cell lymphoma, transmural, large cell, high grade, colon.
A & B.  Round cell tumour, metastatic, mesenteric lymph nodes.
First Comment: As suspected, the mass within the descending colon is a round cell tumour and the histological features are consistent with lymphoma.  Further characterisation of the neoplastic population using immunohistochemistry can be performed on request (lymphoma typing).  No obvious neoplastic tissue is observed within the surgeon-cut tissue borders of the colon.  Therefore complete excision of the mass itself appears to have been achieved.  However, there are foci of neoplastic cells present within the mesenteric lymph nodes.  Therefore the tumour is expected to have potential for further metastasis.
Addendum: The neoplastic cells express CD20 and Pax-5 consistent with a B-cell lymphoma. Based on the mitotic count the tumour is considered to be of histologically high grade.

Case 17

**Clinical History:** Liver, jejunum, duodenum and ileum biopsy, intestinal mass, mesenteric mass. History weight loss, intestinal mass seen on ultrasound and enlarged kidneys. Clinically consistent with lymphoma but FNAs of intestinal mass did not yield anything other than blood and neutrophils. Lymph nodes grossly enlarged. Other intestinal biopsies and liver biopsy from normal appearing tissues.

**Gross description:**

A: Intestinal mass. A beige grey section of tubular tissue measuring 125x45x45mm with a mass measuring 45x45mm. Representative sections taken.

B: Mesenteric LN. A beige-brown irregular wedge of friable tissue measuring 17x11x10mm. Bisected. All tissue used.

C: Mesenteric LN 2. A beige-brown irregular wedge of tissue measuring 17x11x6mm. Bisected. All tissue used.

D: Mesenteric mass. A beige brown grey irregular wedge of tissue measuring 25x16x8mm. Representative sections taken.

E: Liver. A brown irregular wedge of tissue measuring 13x8x7mm. Bisected. All tissue used.

F: Intestines.

Fa: Duodenum. A beige grey irregular piece of tissue measuring 6x5x2mm. Embedded whole. All tissue used.

Fb: Jejunum. A beige grey irregular piece of tissue measuring 6x5x2mm. Embedded whole. All tissue used.

Fc: Ileum. A beige irregular piece of tissue measuring 5x5x2mm. Embedded whole. All tissue used.

**Histological description:**

A. Small intestine (6 sections). The mucosa, submucosa and tunica muscularis are focally extensively effaced, by a non-encapsulated neoplasm. Neoplastic cells are arranged in sheets supported by a fine fibrovascular stroma. Neoplastic cells are round with a moderate amount of cytoplasm and indistinct cell borders. Nuclei are oval, measure approximately 2.5 times the diameter of adjacent erythrocytes, with finely stippled chromatin and one magenta nucleolus.

There is moderate anisocytosis and anisokaryosis. Mitoses average 11 per high power field. The mucosa is extensively ulcerated and in these areas large aggregates of fibrin, neutrophils and karyorrhectic debris are present on the surface. These are admixed with large clusters of bacteria. Neoplastic cells extend beyond the serosa into the adjacent mesentery in multiple areas. Within the mass there are multiple large areas of necrosis with aggregates of fibrin,

karyorrhectic debris and degenerate neutrophils. Within one of the sections through the surgeon-cut tissue borders of the intestinal segment there are large aggregates of neoplastic cells within the tunica muscularis.

B. Mesenteric lymph node (2 sections). The lymph node architecture is diffusely effaced by a neoplastic population of round cells. These exhibit similar features to those described above for (A). Scattered within the supporting stroma there are occasional small lymphocytes.

C. Mesenteric lymph node (2 sections). The lymph node architecture is multifocally extensively effaced by a neoplastic population. The features are as described above for (A). Scattered small aggregates of small lymphocytes are present.

D. Mesenteric mass (1 section). The section comprises a neoplasm, which has undergone extensive necrosis. Within the periphery, there are dense aggregates of round cells, resembling those described above for (A). The majority of the section is composed of large aggregates of eosinophilic cellular debris, admixed with degenerate neutrophils.

E. Liver (2 sections). The hepatic architecture is preserved. Portal areas are well organised. Within the portal areas, there are low numbers of small lymphocytes, admixed with plasma cells. Within periacinar areas, hepatocytes contain small amounts of pale brown pigment. No obvious neoplastic tissue is observed.

Fa. Duodenum (1 section). The mucosal architecture is preserved. Intestinal villi are mildly blunted. Within the lamina propria there is mildly increased fibrous tissue. Populations within the lamina propria are predominantly composed of plasma cells, admixed with fewer small lymphocytes. Numbers of plasma cells are mildly increased. Numbers of intraepithelial lymphocytes are within normal limits (10-20 per x 40 stretch). The submucosa and tunica

muscularis are well organised.

Fb. Jejunum (1 section). The mucosal architecture is overall preserved. Intestinal villi are long and slender. The lamina propria is multifocally moderately expanded by oedema and lymphatic vessels are mildly ectatic. Multifocally within the lumen of crypts there are small aggregates of eosinophilic material, occasionally admixed with small clusters of neutrophils. Within the lamina

propria, numbers of plasma cells and lymphocytes are mildly increased. Numbers of intraepithelial lymphocytes are within normal limits (10-20 per x 40 stretch). The submucosa and tunica muscularis are well organised.

Fc. Ileum (1 section). The mucosal architecture is preserved. Villi are long and slender. Numbers of lymphocytes and plasma cells within the lamina propria are within normal limits. Lacteals are mildly dilated (25-50% of the villous width).

**Histological diagnosis:**

A. Consistent with lymphoma, small intestine.

B & C. Consistent with lymphoma, mesenteric lymph nodes.

D. Consistent with lymphoma, with necrosis, marked, mesenteric mass.

E. No significant changes observed, liver.

Fa. Enteritis, plasmacytic, with fibrosis, multifocal, mild, duodenum.

Fb. Enteritis, plasmacytic and lymphocytic, with oedema, multifocal, mild, jejunum.

Fc. Lacteal dilation, multifocal, mild, ileum.

**Comment:** As suspected clinically, the mass within the intestine is a round cell tumour and the histological features are consistent with lymphoma. There are large areas of necrosis within the mass and these likely account for the cytology findings in this case. The neoplastic cells extend into the adjacent mesentery. Within one of the sections through the surgeon-cut tissue borders of the intestinal segment, there are clusters of neoplastic cells present within the tunica muscularis. Therefore, complete excision cannot be confirmed. Furthermore, there are neoplastic cells present within the mesenteric lymph nodes. The

submitted mesenteric mass has undergone extensive necrosis but likely also represents a mesenteric lymph node, which has been infiltrated by neoplastic cells. Further characterisation of the neoplastic process using immunohistochemistry can be performed on request (lymphoma typing).

No obvious neoplastic tissue is observed within the liver or within the separately submitted biopsies of the duodenum, jejunum and ileum. Within the intestinal biopsies there is evidence of oedema and lacteal dilation, potentially reflecting hypoproteinaemia.

Case 18

**Clinical History:** Jejunal mass, also biopsies of ileum, duodenum, proximal jejunum, caudal mesenteric LN, liver. Mass found on routine health check. Rapid growth. Ultrasound revealed generalised small intestinal muscularis thickening. Cytology indicated mass= mast cell tumour biopsies of enlarged LN (3 cm). And normal liver and small intestine. Differential= high grade lymphoma.

**Gross description:**

A: Intestine mass. A beige section of tubular tissue measuring 200x50x40mm with a mass measuring 50x40mm. Representative sections taken.

B: Duodenum biopsies. 2x beige pieces of tissue measuring up to 2mm diameter. Embedded whole. All tissue used.

C: Proximal jejunum bx. 1x beige piece of tissue measuring up to 2mm diameter. Embedded whole. All tissue used.

D: Ileum bx. 1x beige piece of tissue measuring up to 2mm diameter. Embedded whole. All tissue used.

E: Mesenteric LN. A beige piece of ovoid shaped tissue measuring 11x8x4mm. Bisected. All tissue used.

F: Liver biopsy. 3 beige brown irregular wedges of tissue measuring up to 19x9x5mm. Representative sections taken.

**Histological description:**

A. Jejunum (4 sections). At the site of the mass identified grossly, is a densely cellular, locally infiltrative neoplastic mass which extends from the submucosa and through all layers into the subserosal stroma. Neoplastic cells are arranged in densely packed sheets and cords, supported by small amounts of fibrovascular connective tissue. Individual neoplastic cells are polygonal to round and contain moderate to large amounts of finely vacuolated and finely granular, pale eosinophilic cytoplasm and have distinct cell borders. Nuclei are oval, measure

approximately 1 - 1.5x the diameter of adjacent erythrocytes and contain stippled chromatin with a small nucleolus. Mitoses are less than 1 per 10 high power fields. Occasional dense clusters of small lymphocytes are interspersed with the neoplastic cells. No neoplastic cells are apparent at the proximal or distal surgeon-cut ends of the intestinal segment. There is segmental mucosal

fibrosis at the surgeon-cut edges of the tissue along with mildly increased numbers of lymphocytes and plasma cells within the lamina propria.

B. Duodenum (2 sections). Mucosal villi, lining these full thickness sections of intestine are moderately tall and are lined by tall columnar epithelial cells with expected numbers of goblet cells present. Intraepithelial lymphocytes are present at a frequency of up to 15 cells per high power field stretch. The lamina propria contains expected numbers of lymphocytes and plasma cells representing up to 25% of the total tissue area, with 1-2 cells per intercrypt space. Eosinophils are present at a frequency of up to 5 cells per high power field. Lacteals represent less than 25% of the total villous width. There is mild diffuse fibrosis within the lamina propria. The submucosa and muscularis layers are within normal limits.

C. Proximal jejunum (1 section). Mucosal villi lining the section are tall and slender and lined by tall columnar intact epithelium. The mucosa exhibits similar features to those described for the duodenum above. The submucosa and muscularis layers are within normal limits.

D. Ileum (1 section). This tissue is composed of well organised smooth muscle and submucosa. No mucosal villi are apparent.

E. Mesenteric lymph node (2 sections). The lymph node parenchyma throughout the sections is extensively disrupted and effaced by densely packed sheets of neoplastic cells with the same histological features as those described for the jejunum above.

F. Liver (3 sections). The hepatic parenchyma is diffusely well organised with generally regularly spaced portal triads and central veins present. There are mild proliferations of bile ducts, endothelial cells and fibroblasts within portal triads. Portal veins are of generally expected cross-sectional diameter. Scattered lymphocytes and plasma cells and occasional neutrophils and eosinophils are present within portal triads and are present within central veins. There is

mild diffuse, Ito cell proliferation throughout all zones. There is irregular mild nodular proliferation of hepatocytes. Hepatocytes within mid-zonal to periacinar regions contain moderate amounts of intracytoplasmic, yellow-green granular pigment.

**Histological diagnosis:**

A & E. Mast cell tumour, jejunum with metastasis, mesenteric lymph node.

B & C. Fibrosis, mucosal, diffuse, mild, duodenum and proximal jejunum.

D. No significant lesions, ileum.

E. Hepatitis, portal and periacinar, lymphocytic and plasmacytic, minimal with fibroplasia and biliary hyperplasia, portal, mild, liver.

**Comment:** As anticipated, the mass from the jejunum represents a mast cell tumour that has metastasized to the associated mesenteric lymph node. I can find no evidence of metastasis to the liver, but there are reactive changes within the liver, likely reflecting a response to the effects of the invasive mast cell tumour. Within the small intestine, there are mildly increased amounts of collagenous matrix throughout the lamina propria, this likely reflects a response to previous episodes of pathological inflammation within the small intestine. Indeed, there are increased numbers of lymphocytes within the lamina propria of the jejunum at the ends of the large intestinal segment that was submitted, which would support this pathogenesis. A low grade, chronic waxing / waning

hypersensitivity process could therefore account for these low grade fibrotic changes and the low grade enteritis that is apparent within the larger intestinal segment.. I can find no evidence of a lymphoma within the tissues.

Case 19

**Clinical History:** Mass from ileocaecocolic junction. Hx of weight loss. No evidence of mets on CT.

**Gross description:** A beige-brown section of tubular tissue measuring 110x35x35mm. Representative sections taken.

**Histological description:** Ileocaecocolic junction (5 sections). Diffusely infiltrating the mucosa and submucosa and in a focally extensive area the muscular wall and extending into the adjacent mesenteric adipose tissue is a densely cellular, neoplastic round cell infiltrate. Neoplastic cells are arranged in

dense sheets, infiltrating the pre-formed stroma. Individual neoplastic cells are round to oval with indistinct cell borders and small amounts of pale eosinophilic cytoplasm. Nuclei are round to oval and sometimes indented, with coarsely stippled to slightly clumped chromatin and mostly indistinct nucleoli. Mitoses are up to 3 per high power field. Nuclei are approximately 1.5 times the size of adjacent erythrocytes. In a nodular mass, which may have been a lymph node, the entire mass is composed of sheets of similar neoplastic round cells. In this mass, mitoses are up to 10 per high power field. Nuclei are 2-3 times the size of adjacent erythrocytes. In one of the sections from the surgeon-cut edge, the mucosa is variably infiltrated with a monomorphic population of similar round cells.

**Histological diagnosis:** Lymphoma, ileocaecocolic junction.

**Comment:** Transmurally infiltrating the intestinal wall is a neoplastic round cell infiltrate consistent with lymphoma. In the mucosa and intestinal wall, the mitotic activity is overall low and neoplastic cells are small, consistent with a low grade, small cell lymphoma. However, in a mass lesion, which may have been a lymph node, neoplastic cells are large and display a high mitotic activity.

Therefore, progression to a more aggressive form of lymphoma seems to have occurred. In one of the sections from the surgeon-cut edge, neoplastic cells are diffusely infiltrating the lamina propria suggesting that complete local excision has not been achieved and also suggesting an already quite widespread involvement of the mucosa. Immunophenotyping of the neoplastic cells can be performed upon your request.

Case 20

**Clinical History:** History of vomiting and lethargy. Mass in the Ileo-caeco-colic junction. Biopsies revealed intestinal adenocarcinoma with lymph node involvement.

**Gross description:** A beige grey section of tubular tissue measuring 110x30x25mm. Representative sections taken.

**Histological description:** Ileocaecocolic junction (7 sections including oral and aboral borders). Extending from the ulcerated mucosal surface, extending into the outer muscular layers is a markedly infiltrative, moderate to paucicellular, moderately well demarcated, unencapsulated neoplasm arranged in tubules, dilated acini and nests supported by dense fibrous connective tissue (desmoplasia). Neoplastic cells are polygonal to columnar, with variably distinct cell borders, moderate eosinophilic cytoplasm and a single oval nucleus that contains a single nucleolus and stippled chromatin. There is moderate anisokaryosis and anisocytosis with 20 mitoses observed in ten high power fields. Neoplastic cells frequently surround luminal accumulations of mucin admixed with necrotic debris. Lymphovascular invasion is not observed. The supporting stroma is variably infiltrated by moderate numbers of neutrophils and fewer perivascular lymphocytes, plasma cells and clusters of haemosiderophages. The neoplastic population is not identified within the serosa, the oral or aboral borders.

**Histological diagnosis:** Adenocarcinoma; ileocaecocolic junction.

**Comment:** Present within the sections of intestine there is a highly infiltrative neoplasm extending from the mucosal surface, forming dilated acini, tubules and nests representing an adenocarcinoma of cryptal epithelial origin as previously diagnosed. The neoplasm extends into the outer muscular layer but is not observed to track along the serosa: transcoelomic metastasis is therefore not

suspected on the basis of these sections. Distinct lymphovascular invasion is not observed but I note the history of lymph node involvement, further metastasis cannot be excluded. The neoplasm is not identified within the oral or aboral borders of the submitted segment indicating complete local excision. Given the history of metastasis and the infiltrative nature described, close monitoring of this patient is recommended.

Case 21

**Clinical History:** Duodenal mass.  Masses also seen in liver and spleen.  Swollen lymph nodes on intestines.
Macroscopic Description: Duodenal mass. A beige-brown section of tubular tissue measuring 80x45x30mm with a mass measuring 50x30mm. Margins are inked on receipt of tissue. Representative sections taken.
**Microscopic Description:** Duodenum (4 sections).   Segmentally expanding the duodenum is a marked, transmural infiltrate.  In this thickened segment there is an area of mucosal ulceration in which the mucosa is entirely replaced by abundant eosinophilic necrotic debris admixed with degenerate neutrophils.  Deep to this necrotic region there is a granulation tissue formation.  Extending circumferentially throughout the submucosa and muscularis is a marked, mixed cellular infiltrate.  Cells consist of neutrophils, macrophages as well as an indistinct population of medium-sized round cells.  These cells have variably distinct borders with a scant to moderate amount of pale cytoplasm.  The nuclei are round to oval with stippled chromatin and often a single distinct nucleolus.  There are also several small lymphocytes admixed as well.  Also throughout the wall of the duodenum are prominent hypertrophied fibroblasts and plump, reactive spindle cells.  In many regions the background of spindle cells as well as the macrophages and neutrophils often outnumber the population of infiltrating medium-sized round cells.  There are scattered mitoses within this population, typically with 0-2 present per single high power fields.  Transverse sections of the duodenum from the orad and aborad margins are examined with relatively normal architecture.  Within the lamina propria of the mucosa there is a mild increase in the number of plasma cells present.
**Immunohistochemical Description:**
The neoplastic cells throughout the wall of the duodenum exhibit strong positive cytoplasmic and membranous immunoreactivity for CD3, and typically for CD18 as well.  There are also a population of additional round cells throughout the tissue that also exhibit CD18 positivity.  The neoplastic cells are negative for Pax5 and MUM1.  There are numerous scattered individual cells admixed with the neoplastic cells that exhibit moderate positive nuclear immunoreactivity for Pax5.
**Final Diagnosis:**
T-cell lymphoma, low-grade, intermediate-size, with enteritis, transmural, neutrophilic, histiocytic, and lymphocytic, marked, segmental, duodenum.
[Original diagnosis:  Compatible with lymphoma, intermediate-size, low grade with enteritis, transmural, neutrophilic, histiocytic and lymphocytic, marked, segmental, duodenum.]
**First Comment:**
The submitted section of duodenum contains a markedly thickened segment.  Generally, this is due to a highly mixed infiltrate of cells, predominantly composed of inflammatory cells.  However, there does appear to be a population of uniform intermediate sized lymphocytes, and I am concerned that these represent an intermediate cell lymphoma.  (This is also concerning in light of the provided history that additional nodules are seen in the liver and spleen with enlarged lymph nodes.)  Additional immunohistochemical would be helpful in this case to try to highlight the lymphocyte population throughout the wall of the duodenum.  Due to the highly mixed nature of the infiltrate, the noted inflammatory cells often obscure the suspected neoplastic population of lymphocytes.  Please call if you would like to request these additional immunohistochemical stains (panel of 4 antibodies).
Addendum: Results of immunohistochemical staining indicate that the infiltrating lymphocytes throughout the wall of the duodenum are consistent with T-cells, indicative of a T-cell lymphoma.  These neoplastic cells are admixed with frequent macrophages, plasma cells, and neutrophils, as previously described.  I am somewhat surprised that the mitotic rate is quite  low in this case, as this is a highly invasive population of lymphocytes and appears somewhat aggressive.  The wall of the duodenum is highly inflamed and there are frequent inflammatory cells admixed with the neoplastic population. 
The history indicates masses in the liver and spleen, and though those could represent additional lymphoma lesions, it is not possible to predict within additional diagnostic tissue from these organs.  

Case 22

**Clinical History:** No history given.

**Gross description:** A beige section of tubular tissue measuring 110x45x35mm, with a mass measuring 45x30mm. Representative sections taken.

**Histological description:** Small intestine (5 sections). Arising within the mucosa and extending through the submucosa, muscularis and out to the serosa is a poorly demarcated, highly infiltrative, neoplastic mass that appears to have been fully resected with this segment of intestine. The neoplastic cells are

arranged in densely cellular sheets supported by a scant fibrovascular stroma. Individual neoplastic cells are round with relatively discrete cell borders and contain scant to moderate amounts of eosinophilic cytoplasm. Nuclei are round to oval, with frequent, slightly cleaved or irregular nuclei. They contain stippled to clumped chromatin and often have 1-3 variably sized nucleoli. Anisocytosis and anisokaryosis are moderate, and in many regions there are up to 5

-10 mitotic figures per single high power field. The affected areas of mucosa are often ulcerated and replaced by both the neoplastic cells as well as abundant accumulations of fibrin aggregates and hypereosinophilic necrotic debris. Sections of tissue from the orad and aborad margins of the tissue show no evidence of the neoplastic cells present. Throughout the lamina propria,

there is a minimal to mild infiltrate of small lymphocytes which represent up to 50% of the villus width and slightly separate the crypts.

**Histological diagnosis:** Lymphoma, large cell, intermediate grade, with enteritis, lymphocytic, mild, chronic, small intestine.

**Comment:** The mass within the submitted segment of intestine is confirmed as an intermediate grade lymphoma. This is expected to be a progressive disease and is likely to affect additional lymph nodes and viscera. In the marginal sections of tissue examined, there is no evidence of the neoplastic population suggesting that the entire mass has been resected. Continued monitoring

of lymph nodes and additional viscera would be warranted.

Case 23

**Clinical History:** Duodenal obstruction (1 month clinical signs). Asthma- steroids. Ex lap- end to end anastomosis of duodenum. 1) Excision of mural duodenal mass (?) causing complete duodenal obstruction. Lesion marked with suture. 2) Local lymph node. 3) Jejunal lymph node.

**Gross description:**

A- 1. A beige grey section of tubular tissue measuring 60x25x15.. Representative sections taken.

B- 2. A beige irregular piece of tissue measuring 3x2x2mm. Embedded whole.All tissue used.

C- 3. A beige irregular wedge of tissue measuring 3x2x2mm. Embedded whole. All tissue used.

**Histological description:**

A. Duodenum (4 sections). Arising from the mucosal epithelium is a poorly demarcated, unencapsulated and highly infiltrative, neoplastic mass that extends through the submucosa and muscularis and out to the serosa. The mass is composed of multiple, variably sized, irregular tubular structures and occasional small nests, supported by a moderate amount of fibrous to desmoplastic stroma. Individual neoplastic cells are polygonal to columnar with indistinct borders and a moderate amount of eosinophilic cytoplasm. Nuclei are oval with stippled

chromatin and typically 1-2 large, prominent nucleoli. Anisocytosis and anisokaryosis are moderate, and there are 17 mitotic figures per 10 high power fields. Frequently admixed amongst the neoplastic cells are small aggregates of lymphocytes and occasional plasma cells. The neoplastic tubules are often mildly dilated and contain aggregates of hypereosinophilic necrotic material admixed with degenerate inflammatory cells. Neoplastic cells extend out into

the attached mesentery and are surrounded by a moderate infiltrate of lymphocytes. Transverse sections from the proximal and distal surgeon-cut edges of the duodenum are examined with no evidence of neoplastic cells present. In both segments, there is mild blunting and widening of the villi, and there is a consistent, mild to moderate infiltrate of lymphocytes arranged in a band-like formation at the base of the villi. Crypts are often mildly dilated and

contain a small amount of eosinophilic proteinaceous material and occasional necrotic cells.

B & C. Local and jejunal lymph nodes (2 sections). These sections of tissue exhibit varying degrees of crush artefact, most prominent within the tissue labelled as “local lymph node.” Both have poorly formed lymphoid follicles and appear to have a mild increase in plasma cells present. There is mild to moderate sclerosis of the supporting stroma, most prominent in the “local” lymph node. There is no overt evidence of neoplastic cells in either of the lymph node sections, however, interpretation is hampered by the described crush artefact.

**Histological diagnosis:**

A. Adenocarcinoma, duodenum.

B & C. Reactive changes with stromal sclerosis, mild, chronic, local and jejunal lymph nodes.

**Comment:** As suspected, there is a mural mass located within the duodenal wall. Although this mass has arisen from the mucosal epithelium, the bulk of the neoplastic cells are located within the submucosa and muscularis, and they extend out into the attached mesentery. These findings are consistent with an intestinal adenocarcinoma. These neoplasms can be both locally aggressive and have the potential to metastasise. Although there is no evidence of metastasis

within the two submitted sections of lymph node, continued monitoring of lymph nodes as well as additional organs would be warranted. No neoplastic cells are seen in the margins of the submitted tissue, indicating complete resection of the mass.

Case 24

**Clinical History:** Mass in ileocaecal junction removed.

**Gross description:** A beige grey section of tubular tissue measuring 80x45x35mm. Representative sections taken.

**Histological description:** Ileocaecal junctoin (4 sections). The mucosal epithelium has undergone segmental neoplastic transformation with neoplastic cells extending from the ulcerated mucosal surface and through all layers of the intestinal wall, into subserosal stroma and are present partly disrupting the

associated lymph node. Neoplastic cells are arranged in tubular structures, nests and cords and are supported by abundant dense collagenous matrix. Individual neoplastic cells are cuboidal to polygonal and contain moderate amounts of eosinophilic cytoplasm with indistinct cell borders. Nuclei are moderately large, oval to round and contain vesicular chromatin with 1-2 prominent large nucleoli. Mitoses are less than 1 per high power field. Where ulcerated, extensive accumulations of degenerate are present on the exposed submucosal surface. No neoplastic cells are apparent at the proximal or distal surgeon–cut ends of the sample.

**Histological diagnosis:** Carcinoma, transmural with lymph node metastasis, ileocaecal junction.

**Comment:** This mass represents a carcinoma that has infiltrated from the ulcerated mucosal surface and through all layers of the intestinal wall, with metastasis to the associated lymph node. Therefore although the mass does appear to have been fully excised, with no neoplastic cells at the proximal or distal surgeon-cut edges of the intestinal segment, there would be high potential for further metastasis from this site.

Case 25

**Clinical History:** 1 week post endoscopy. Developed anorexia, vomiting and abdomen pain. Surgical explorationrevealed necrotic thickened area of jejunum resected performed. Abdominal fluid taken for cuture and cytology ?bowel ?? Is this a mass

**Gross description:** A beige grey section of tubular tissue measuring 85x30x20mm. Representative sections taken.

**Histological description:** Jejunum (7 sections). Infiltrating throughout the mucosa and extending into the underlying submucosa are densely packed aggregates of neoplastic cells. Neoplastic cells are arranged in sheets and cords, supported by mildly increased amounts of collagenous matrix. Individual

neoplastic cells are round, contain small amounts of eosinophilic cytoplasm and have distinct cell borders. Nuclei are round and contain densely stippled chromatin and measure approximately the same diameter as adjacent erythrocytes. Mitoses are less than 1 per high power field. In some areas the mucosa is ulcerated, with moderate accumulations of necrotic cellular debris on

the exposed submucosal surface. In some areas, there is full thickness ulceration of the intestinal wall with dense accumulations of necrotic cellular debris, proteinaceous exudate and degenerate neutrophils on the exposed serosal surface. Neoplastic cells extend within the mucosa to the surgeon-cut ends of the intestinal segment.

**Histological diagnosis:** Lymphoma, small cell, low grade; with ulceration, full thickness, locally extensive, marked with peritonitis, neutrophilic, marked, jejunum.

**Comment:** Consistent with the clinical findings, there is a lymphoma which has infiltrated widely throughout the intestinal mucosa and which extends to the ends of the intestinal segment. This has likely then predisposed to bacterial overgrowth, which has then resulted in the extensive ulceration and rupture of the intestinal segment. Given these findings, there would be significant potential

for further invasion of the neoplastic lymphocytes to other intestinal sites and this is likely to ultimately progress to lymph nodes and to other internal organs. The neoplastic lymphocytes are small and have a very low mitotic activity, indicating a low grade to indolent lymphoma type that has likely been present for some time.

Case 26

**Clinical History:** Colon (distended). Suspicion of tumour. US- marked asymmetrical thickening of colonic wall with loss of layering.

**Gross description:** Colon Distended. A beige grey section of tubular tissue measuring 90x30x25mm. Representative sections taken.

**Histological description:** Colon (4 sections). Infiltrating the lamina propria, submucosa and tunica muscularis, there is a non-encapsulated neoplasm. Neoplastic cells are arranged in sheets within a fine fibrovascular stroma. Neoplastic cells are round with a moderate amount of eosinophilic cytoplasm and indistinct cell borders. Nuclei are oval, measure approximately 2-2.5 x the diameter of adjacent erythrocytes, with finely stippled chromatin and 1 magenta nucleolus. There is moderate anisocytosis and anisokaryosis. Mitoses average 11 per high power field. Within sections through the surgeon-cut tissue borders of the colon there are well organised nodular aggregates of small and intermediate sized lymphocytes within the deep lamina propria and superficial submucosa.

**Histological diagnosis:** Round cell tumour, consistent with lymphoma, colon.

**Comment:** As seen clinically, there is thickening of the colonic wall due to infiltration by a neoplastic process. The neoplastic cells exhibit features consistent with large cell lymphoma. Further characterisation of the neoplastic population using immunohistochemistry can be performed on request (lymphoma typing). In the sections through the oral and aboral margins of the colonic segment there are nodular aggregates of lymphocytes. These are well organised and are interpreted to represent hyperplastic mucosal lymphoid tissue. No obvious infiltrative round cell population is observed in the margins. Lymphoma may however be multifocal within the intestine and further masses could arise. Furthermore there is potential for metastasis from this site.

Case 27

**Clinical History:** Large intestinal mass removed.

**Gross description:** A beige grey section of tubular tissue measuring 125x55x40mm, with a mass measuring 558x40mm. Representative sections taken.

**Histological description:** Small intestine (5 sections, 2 levels including shaved oral and aboral borders). Arising from and circumferentially elevating the overlying mucosa, with subsequent partial occlusion of the intestinal lumina, is a markedly infiltrative, unencapsulated, moderately well demarcated, unencapsulated neoplasm arranged variably in tubules, acini and infiltrative nests, supported by dense fibrous connective tissue (desmoplasia). Neoplastic cells are variably polygonal to cuboidal, with indistinct cell borders, moderate eosinophilic cytoplasm and a single round to irregular oval nucleus. Nuclei contain large nucleoli and stippled chromatin. There is moderate

anisocytosis, moderate to marked anisokaryosis and 14 mitoses are identified within 10 high power fields. Frequently, neoplastic cells are individually shrunken, hypereosinophilic, with pyknotic nuclei (necrosis). Neoplastic acini and tubules often contain luminal accumulations of sloughed neoplastic cells, necrotic debris and lightly basophilic amorphous material (mucin). The

neoplastic population extends transmurally, to less than 1mm from the serosal surface, however infiltration of the adjacent mesenteric adipose and tracking along the serosal surfaces are not identified. Neoplastic cells are not observed within the oral or aboral borders.

**Histological diagnosis:** Adenocarcinoma, small intestine.

**Comment:** The submitted small intestinal mass is a tumour of cryptal epithelial origin, that is markedly infiltrative, extending transmurally but appears removed from the planes of tissue examined. The neoplastic population is accompanied by severe desmoplasia, resulting in constriction of the intestinal lumen. Distinct lymphovascular invasion is not observed, however metastasis from this

site to local draining lymph nodes and further distant sites cannot be excluded. The neoplastic population is not identified tracking along the serosal surface, and therefore transcoelomic metastasis is not suspected, but cannot totally be excluded. The neoplastic population is not identified within the shaved oral and aboral borders, indicative of a full excision of this neoplasm. Despite this apparent full excision, periodic monitoring of this patient is recommended.

Case 28

**Clinical History:** Ileocolic mass. Acute weight loss and intestinal mass. Cytology suggestive of carcinoma. Marked regional lymphadenopathy at surgery. Resection and end to end anastomosis performed. Please assess surgical margins.

**Gross description:** A beige section of tubular tissue measuring 100x30x20mm. Representative sections taken.

**Histological description:** Ileocolic mass (3 sections). Arising within the intestinal mucosa and multifocally infiltrating the submucosa and tunica muscularis, there is a poorly demarcated neoplasm. Neoplastic cells are

arranged in cords and form tubular structures, supported by abundant fibrous stroma. Neoplastic cells are cuboidal to polygonal with a moderate amount of eosinophilic cytoplasm and indistinct cell borders. Nuclei are oval with finely stippled chromatin and 1-2 magenta nucleoli. There is moderate anisocytosis and anisokaryosis. 11 mitoses are observed in 10 high power fields. Within the lumen of neoplastic tubules there are aggregates of karyorrhectic debris and

mucinous material. The intestinal mucosa is multifocally ulcerated. Within the adjacent mesentery neoplastic cells occasionally form large clusters within the lumen of lymphatic vessels. Within the section through the surgeon-cut tissue borders of the colon there are occasional tubular structures within the submucosa which are formed by cuboidal to polygonal epithelial cells similar to the neoplastic cells. These tubules are surrounded by large numbers of

lymphocytes forming nodular aggregates. Similar epithelial cells are present in the section through the ileal border.

**Histological diagnosis:** Carcinoma, ileocolic mass.

**Comment:** Consistent with the previous cytology result, the mass within the intestine is a carcinoma. This tumour exhibits extensive infiltration into the intestinal wall. Furthermore there are clusters of neoplastic cells present within the lymphatics in the mesentery. Therefore this tumour would be expected to have significant potential for metastasis from the site. In the sections through the

surgeon-cut tissue borders of the colon and ileum there are tubular structures within the submucosa, resembling the neoplastic population. I considered the possibility that these could represent herniated crypts as they are associated with prominent lymphoid follicles. However the epithelial cells lining the tubules are not well differentiated and are interpreted to likely represent neoplastic cells. Therefore the tumour has likely been incompletely excised or the neoplastic foci in the margins represent lymphatic dissemination. In either case there is potential for local recurrence.

Case 29

**Clinical History:** No history given with submission received. LN biopsy. Bowel biopsy.

**Gross description:**

A: A beige grey section of tubular tissue measuring 85x30x20mm with a mass measuring 30x20mm. Representative sections taken.

B: LN mesenteric. A beige irregular wedge of tissue measuring 7x3x2mm. Embedded whole. All tissue used.

C: Bowel. A beige irregular wedge of tissue measuring 5x3x3mm. Bisected. All tissue used. Tissue fixed further prior to processing.

**Histological description:**

A. Small intestine (3 sections including shaved surgeon-cut tissue borders). Present within the intestinal propria, extending transmurally to the serosal surface, is a markedly infiltrative, moderately well demarcated, unencapsulated, densely cellular neoplasm arranged in sheets and thin cords, supported by the pre-existing stroma. Neoplastic cells are large, round, with distinct cell borders, abundant eosinophilic cytoplasm that contains scant to abundant, small, basophilic granules, and a single round to oval nucleus. Nuclei contain indistinct to 1 small nucleolus and stippled chromatin. Nuclei measure approximately 1-1.25 times the size of adjacent eosinophils. There is mild to moderate anisocytosis and anisokaryosis, with 4 mitoses identified in 10 high power fields. Scattered throughout the neoplasm, there are significant numbers of eosinophils.

The supporting stroma is multifocally expanded by moderate to high numbers of extravasated erythrocytes (haemorrhage). The neoplastic population is not overtly identified within the shaved surgeon-cut tissue borders.

B. Mesenteric lymph node (1 section 2 levels). This section lacks natural borders and is composed of a loosely arranged fibrovascular connective tissue that is subtotally replaced by a neoplastic population arranged in sheets, with similar histological features to those described above (A). There is mild to moderate anisocytosis and anisokaryosis with 2 mitoses identified in 10 high power fields. The neoplastic population is separated by moderate numbers of eosinophils. At the edge of the section, there are moderate to high numbers of small, crushed

lymphocytes (remnant lymph node).

C. Small intestine (2 sections). These sections are lined by generally slender and uniformly shaped villi that are lined by an intact single layer of tall columnar epithelium. The villi contain a central lacteal that comprises less than 25% of the villous width. The epithelium contains expected numbers of intraepithelial lymphocytes (up to 20 per times 40 high power field). The propria contains perpendicularly orientated crypts separated by 1-2 fibroblasts as expected. The

propria contains mildly increased numbers of lymphocytes and plasma cells, comprising up to 30% of the area of a x40 HPF. Scattered throughout the propria there are moderate numbers of eosinophils (up to 20 per x40 HPF). The submucosa, muscular layers and plexi are well organised.

**Histological diagnosis:**

A. Mast cell tumour, small intestine.

B. Mast cell tumour, metastasis, mesenteric lymph node.

C. Enteritis, lymphocytic plasmacytic, eosinophilic, chronic, multifocal, mild to moderate, small intestine.

**Comment:** The submitted intestinal mass is a mast cell tumour. The neoplastic population is locally aggressive, extending transmurally to the serosal surface. The neoplastic population is not identified within the shaved surgeon-cut tissue borders, however, there is evidence of metastasis to the sampled mesenteric lymph node. Despite the apparent full excision of the primary intestinal mass, close monitoring of this patient is warranted because further local and distinct

metastasis may be anticipated in this case. The additional sample of small intestine is inflamed with infiltration by a predominantly lymphoplasmacytic population, admixed with moderate numbers of eosinophils. Ulceration and

fibrosis are not identified in this sample. The cause of the inflammation is not apparent; bacteria, endoparasites and neoplasia are not identified in the sample . Given the reported presence of an intestinal mast cell tumour however, the inflammation may well be secondary to local release of cytokines.

Case 30

**Clinical History:** Diagnosed lymphoma of intestinal mass. FNA for months ago - started COAP chemo then changed to pred and chlorambucil. Weight loss - mass reduced but not resolved. Surgery - mass at ileocolic junction and biopsy, biopsy local lymph node that was enlarged and biopsy duodenum.

**Gross description:**

A: intestinal mass. A beige grey section of tubular tissue measuring 40x25x17mm. Representative sections taken.

B: Duodenum. A beige grey irregular piece of tissue measuring 3x3x3mm. Embedded whole. All tissue used.

C: lymph node. A beige grey irregular wedge of tissue measuring 5x3x3mm. Embedded whole. All tissue used.

**Histological description:**

A. Ileo-colic junction (5 sections). Within the mesentery and extending into the adjacent tunica muscularis of the caecum, there is non-encapsulated neoplasm. Neoplastic cells are arranged in sheets, supported by a pre-existing fibrous stroma. Neoplastic cells are round with a moderate amount of eosinophilic cytoplasm and indistinct cell borders. Nuclei are round to oval, measure

approximately 2.5x the diameter of adjacent erythrocytes, with finely stippled chromatin and one prominent magenta nucleolus. There is moderate anisocytosis and anisokaryosis. Mitoses average 7-8 per high power field. Within the mass there are multiple large areas of necrosis. In some areas the mass is bordered by a thin rim of compressed lymph node tissue. Within a section through the proximal border of the ileum, intestinal villi are mildly blunted. Within the lamina propria there are moderately increased numbers of plasma cells and lymphocytes. Lymphocytes within these sections are of small size. The submucosa and tunica muscularis are well organised. Within a section through the surgeon-cut tissue border of the colon, the mucosal architecture is preserved. Crypts are well organised. Numbers of lymphocytes and plasma cells within the lamina propria are mildly increased. There are occasionally mildly dilated crypts. The submucosa and tunica muscularis are well organised.

B. Duodenum (1 section). The mucosal architecture is overall preserved. Villi are moderately blunted. Within the lamina propria crypts are separated by mildly increased fibrous stroma. Numbers of plasma cells within the lamina propria are mildly increased and are admixed with fewer lymphocytes. The submucosa and tunica muscularis are well organised.

C. Mesenteric lymph node (1 section). The lymph node architecture is mildly distorted. Within the cortex there are multiple follicles with prominent germinal centres. Within medullary sinuses there are large numbers of macrophages containing pale brown pigment. Within medullary sinuses there are occasional large round cells which exhibit prominent nucleoli. Occasionally these exhibit mitotic figures.

**Histological diagnosis:**

A. Consistent with lymphoma, mesenteric lymph node, adjacent to caecum.

B. Enteritis, plasmacytic, with fibrosis, multifocal, mild, duodenum.

C. Follicular hyperplasia, with round cell infiltrate, multifocal, mesenteric lymph node.

**Comment:** Within the sample from the ileo-colic junction, there is a neoplastic process, which appears to arise within a mesenteric lymph node. Neoplastic cells infiltrate the deeper aspects of the tunica muscularis of the adjacent caecum. However no obvious neoplastic tissue is observed within the mucosa of the caecum or the adjacent ileum or colon. Therefore it appears that this tumour has

likely arisen within a mesenteric lymph node. Consistent with the previous FNA findings, the most likely diagnosis is lymphoma. The cells are of large size and based on the mitotic count, the tumour would be of histologically intermediate grade. No obvious neoplastic tissue is observed within the separately submitted sample from the duodenum. Within the separately submitted sample of mesenteric lymph node there are occasional large round cells present within the sinuses. These do not form a confluent mass but the cells resemble the neoplastic population within the lymph node adjacent to the caecum. Therefore early metastasis to this lymph node appears likely.

Case 31

**Clinical History:** Resect large intestinal mass - ileum. also biopsy enlarged mesenteric LN. Please check intestinal margins for evidence neoplasia.

**Gross description:**

A- 1. A beige grey section of tubular tissue measuring 130x50x30mm, with a mass measuring 50x40mm. Representative sections taken.

B- Mesenteric L Node. A beige-brown irregular wedge of tissue measuring 10x5x3mm. Embedded whole. All tissue used.

**Histological description:**

A. Ileal excision specimen (4 sections). Two of these sections are multinodular and hypercellular with no mucosa apparent. The neoplastic cells are in relatively dense sheets, supported by a pre-existing collagenous stroma. The cells themselves are round to irregular and have moderately distinct cell margins and sparse amounts of eosinophilic cytoplasm. Nuclei are round to oval and are approximately 1-1.5x the size of adjacent erythrocytes. Chromatin is coarsely

clumped and many of the cells contain at least one small hyperchromatic nucleolus. Mitoses in this cell population are 31 per 10x400 fields. In these sections although there is no extant mucosa there is an area of accumulated serocellular debris and necrosis extending into the subjacent lamina propria which is delineated by seams of immature fibrous tissue (fibroplasia).

Elsewhere there are focal accumulations of fibrin and necrotic debris with moderate aggregates of neutrophils; neutrophils are also exocytosing from blood vessels. Two of these sections comprise excision edges of the specimen. In both these sections there is moderately prominent MALT forming nodules within the submucosa and extending into the mucosa. No overt neoplastic cells are observed in these sections however.

B. Mesenteric lymph node (1 section). Within this lymph node are prominent germinal follicles with broad germinal centres. Between these germinal follicles are mature and well-differentiated lymphocytes and plasma cells. There are also moderate accumulations of neutrophils. Medullary sinuses are mildly expanded by proteinaceous fluid, also erythrocytes.

**Histological diagnosis:**

A. Lymphoma, small cell, low grade, ileum.

B. Lymphoid hyperplasia, marked, mesenteric lymph node.

**Comment:** The histological appearance of the mass confirms presence of a lymphoma at the site. This tumour is composed of small neoplastic cells of a low grade given their mitotic activity. The tumour would be expected to be indolent and slowly progressive, eventually spreading from the primary site.

No neoplastic cells are observed within two sections of oral and aboral excision edges although mucosa-associated lymphoid tissue is prominent in these sections suggesting hyperplasia at least. In the enlarged mesenteric lymph node there is again hyperplasia of the lymphoid tissue but no overt neoplastic cells are observed.

Case 32

**Clinical History:** Caeco-colic mass and hyperplastic lnn removed with ileo-colonectomy.

**Gross description:** A beige grey section of tubular tissue measuring 80x50x25mm with a mass measuring 25x20mm. Representative sections taken.

**Histological description:**

1. Caecocolic junction (3 sections). Expanding the ulcerated mucosal surface, infiltrating transmurally, is a moderately well demarcated, densely cellular, unencapsulated neoplasm arranged in sheets, supported by the pre-existing, mildly inflamed stroma. Neoplastic cells are large, round, with indistinct cell borders, moderate to abundant eosinophilic cytoplasm that contains a single round nucleus. Nuclei measure greater than 2x the size of adjacent erythrocytes, contain 1-2 nucleoli and finely stippled chromatin. There is moderate anisocytosis and anisokaryosis. Up to 12 mitoses are identified within a single high power field. Individual neoplastic cells are frequently shrunken, hypereosinophilic, with pyknotic nuclei. Scattered throughout the neoplasm and multifocally forming clusters, there are low to moderate numbers of

neutrophils, fewer lymphocytes and plasma cells. The neoplasm is often separated by necrotic debris admixed with haemorrhage. Neoplastic cells are not overtly identified within the shaved surgeon-cut tissue borders, which otherwise appear viable.

2. Lymph node (1 section). Expanding the subcapsular sinuses, extending to the paracortical regions, there is a focally infiltrative, unencapsulated, densely cellular neoplasm, with similar histologic features to those described above (1). The neoplastic population is separated by low numbers of neutrophils. The adjacent sinuses are moderately ectatic. Cortical regions are otherwise expanded by lymphoid follicles that are well organised.

**Histological diagnosis:**

1. Round cell neoplasm (see comment), caecocolic junction.

2. Round cell neoplasm, metastasis, lymph node.

**Comment:**

The examined caecocolic junction contains a neoplasm of round cell origin. The neoplastic population exhibits morphology most compatible with lymphoma, however immunohistochemistry is recommended to confirm this diagnosis and immunophenotype the population (lymphoma panel, available on request). The neoplasm has a high mitotic index and therefore a rapidly progressive disease course may be encountered. There is evidence of metastasis to the sampled lymph node, therefore further local and distant metastasis may be encountered in this case. Close monitoring of this patient is warranted.
